# *GIPAW* Pseudopotentials of *d* Elements for Solid-State NMR

**DOI:** 10.3390/ma15093347

**Published:** 2022-05-06

**Authors:** Christian Tantardini, Alexander G. Kvashnin, Davide Ceresoli

**Affiliations:** 1 Department of Chemistry, UiT The Arctic University of Norway, P.O. Box 6050 Langnes, N-9037 Tromsø, Norway; 2 Institute of Solid State Chemistry and Mechanochemistry SB RAS, 630128 Novosibirsk, Russia; 3 Skolkovo Institute of Science and Technology, Bolshoy Boulevard 30, bld. 1, 121205 Moscow, Russia; a.kvashnin@skoltech.ru; 4 CNR-SCITEC, c/o Dipartimento di Chimica, Università degli Studi di Milano, Via Golgi 19, 20133 Milano, Italy

**Keywords:** *GIPAW*, *d* elements, NMR, chemical shift, quadrupolar coupling constant

## Abstract

Computational methods are increasingly used to support interpreting, assigning and predicting the solid-state nuclear resonance magnetic spectra of materials. Currently, density functional theory is seen to achieve a good balance between efficiency and accuracy in solid-state chemistry. To be specific, density functional theory allows the assignment of signals in nuclear resonance magnetic spectra to specific sites and can help identify overlapped or missing signals from experimental nuclear resonance magnetic spectra. To avoid the difficulties correlated to all-electron calculations, a gauge including the projected augmented wave method was introduced to calculate nuclear resonance magnetic parameters with great success in organic crystals in the last decades. Thus, we developed a gauge including projected augmented pseudopotentials of 21 *d* elements and tested them on, respectively, oxides or nitrides (semiconductors), calculating chemical shift and quadrupolar coupling constant. This work can be considered the first step to improving the ab initio prediction of nuclear magnetic resonance parameters, and leaves open the possibility for inorganic compounds to constitute an alternative standard compound, with respect to tetramethylsilane, to calculate the chemical shift. Furthermore, this work represents the possibility to obtain results from first-principles calculations, to train a machine-learning model to solve or refine structures using predicted nuclear magnetic resonance spectra.

## 1. Introduction

The application of NMR spectroscopy to rigid or semi-rigid solid samples allows the study a plan of systems as bio-molecules with high molecular weight, polymers, perovskites (e.g., solar cells absorbers) and cements in chemistry and chemical sciences. NMR is the oscillatory response of nuclei with non-zero nuclear spins (total angular momentum) immersed in an external field $\left(B_{0}\right)$. The presence of **B**${}_{0}$ removes the degeneracy of nuclear spins, leading to the energy difference:

(1) $$\Delta E=\gamma \hbar \left(1-\sigma \right)B_{0}$$

where γ is the gyromagnetic ratio; and σ is the chemical shielding around a nucleus, which is a characteristic of a specific isotope. Thus, the chemical structure can be revealed by NMR frequencies that are significantly affected by γ and σ. NMR frequencies are reported as a chemical shift (δ), which is the fractional difference between the frequency of a particular nucleus and a standard compound such as tetramethylsilane (TMS). If NMR seems to be, abstractly, the best way to determine chemical structure, the NMR frequencies are strictly anisotropic, being dependent on the relative orientation between $B_{0}$ and a sample, with the consequent generation of internuclear couplings and quadrupolar couplings. Actually, the quadrupolar coupling constant $\left(C_{Q}\right)$ can be estimated as

(2) $$C_{Q}=\frac{eQV_{zz}}{h}$$

where eQ is the electric quadruple moment, $V_{zz}$ is the potential of electric field $B_{0}$ along *z*-axes, and *h* is the Planck constant. Thus, these anisotropic interactions need to be partially averaged through the molecular rotations, and measurement of motionally averaged NMR spectra and induced nuclear spin relaxation reveals the geometries and rates of motion. Furthermore, the nuclear magnetic resonance (NMR) signal is orders of magnitude lower in frequency than the microwave, infrared and ultraviolet frequencies employed in rotational, vibrational and electronic spectroscopes. This is due to the low population difference between nuclei with removed degeneracy and those in the ground state, causing low signal-to-noise ratios along the spectra. In solid-state NMR, low signal-to-noise ratio is accentuated by the presence of acoustic phonon deformation potential (ADP) scattering and optical phonon branches. They can be responsible for electron-phonon coupling, which can, alternatively, affect the population difference. Thus, the development of NMR is focused on the increasing of experimental sensitivity. This can be carried out through increasing the intensity of an applied magnetic field, with the consequent increase in ΔE, but it is very expensive. Or it can be carried out by recording NMR spectra in the domain following a radio-frequency pulse and obtaining the spectrum by Fourier transformation rather than by sweeping the frequency and measuring absorption or emission in classical spectra. Fourier transformation NMR spectroscopy increases by one order of magnitude and opens the door to multidimensional NMR spectroscopy. In spite of its sensitivity, the interpretation of NMR spectra can be less intuitive than microscopy or diffraction data, because structural information is encoded in frequency spectra rather than spatial density maps. The frequency peaks need to be assigned to individual atoms, which can be a significant challenge. However, the multitude of peaks in NMR spectra represent an exquisite chemical fingerprint of molecules, thus making NMR spectroscopy of great use to chemists. Thus, computational methods are increasingly used to support interpreting, assigning and predicting the solid-state NMR spectra of materials. Furthermore, density functional theory (DFT) gave excellent results with gauges including atomic orbitals [1] (GIAO) for soft-matter NMR; this approach cannot be applied to solid-state NMR, because all-electron calculations are not performable due to required computational resources and the necessity to preserve translation symmetry in solids. Thus, in the framework of plane-waves DFT, a gauge including the projected augmented wave [2,3] (*GIPAW*) method was introduced to calculate nuclear resonance magnetic (NMR) parameters in solids, avoiding all-electron calculations. In the *GIPAW* approach [4,5], a uniform magnetic field is applied using boundary conditions, a *periodic magnetic field* with a finite wavelength $r_{G}$ is the gauge origin and is subsequently extrapolated in the limit $r_{G}\to 0$ to compute the chemical shielding. This formalism was seen to manage the numerical instabilities associated with the summation of two divergent terms and with the generalized gradient approximation exchange-correlation functional (the method of choice for condensed-matter simulations) to perform accurate results [6,7,8,9,10,11,12,13,14,15]. In this work we have developed *GIPAW* pseudopotentials for the elements of first, second and third rows of *d* elements excluding La, which is considered as a part of the Lanthanides. These pseudopotentials were tested on the oxides or nitrides optimizing the crystal structures and, subsequently, we calculated the NMR parameters. The developing of these pseudopotentials was carried out to increase the number of compounds for which NMR parameters can be calculated.

## 2. Theoretical Background

In plane-waves DFT the all-electron potential of an atom is substituted by a mathematical object, the so-called pseudopotential. The all-electron wave functions are substituted by pseudo-wavefunctions that eliminate the core states and describe only the chosen valence pseudo-wavefunctions. This limits the generation of a pseudopotential with a specific configuration. Actually, there are different types of pseudopotential: norm-conserving [16], ultrasoft [17] and projected augmented wave (PAW) [18]. The last introduces a linear operator

(3) $$\Gamma =1+\sum_{R,n}(|\phi_{R,n}\rangle -|{\tilde{\phi }}_{R,n}\rangle )\langle {\tilde{p}}_{R,n}|$$

that converts the pseudo-wavefunctions $|{\tilde{\phi }}_{R,n}\rangle$ to all-electron wavefunctions $|\phi_{R,n}\rangle$. In addition, $\langle {\tilde{p}}_{R,n}|$ is a set of projectors such that $\langle {\tilde{p}}_{R,n}|{\tilde{\phi }}_{R,n}\rangle =\delta_{R,R^{\prime }}\delta_{n,m}$.

Thus, it is possible to introduce in the Blöchl formalism [19] a field dependent transformation operator $\Gamma_{B}$ that restores the translational invariance.

(4) $$\begin{array}{cc} \Gamma_{B}= & 1+\sum_{R,n}e^{(i/2c)r\cdot R\times B}(|\phi_{R,n}\rangle -|{\tilde{\phi }}_{R,n}\rangle )\\ \; & \langle {\tilde{p}}_{R,n}|e^{-(i/2c)r\cdot R\times B} \end{array}$$

Such re-formalism is called gauge including projected augmented wave (*GIPAW*) [2,3] and satisfies the translation relation $|\Psi \rangle =\Gamma_{B}|\tilde{\Psi }\rangle$. The *GIPAW*pseudo-operator $\bar{O}=\Gamma_{B}^{+}O\Gamma_{B}$, corresponding to a local or a semilocal operator *O*, is given by

(5) $$\begin{array}{cc} \bar{O}= & O+\sum_{R,n,m}e^{(i/2c)r\cdot R\times B}|{\tilde{p}}_{R,n}\rangle \\ \; & \times (\langle \phi_{R,n}|e^{-(i/2c)r\cdot R\times B}Oe^{(i/2c)r\cdot R\times B}|\phi_{R,m}\rangle \\ \; & -\langle {\tilde{\phi }}_{R,n}|e^{-(i/2c)r\cdot R\times B}Oe^{(i/2c)r\cdot R\times B}|{\tilde{\phi }}_{R,m}\rangle )\\ \; & \times \langle {\tilde{p}}_{R,m}|e^{(i/2c)r\cdot R\times B} \end{array}$$

The *GIPAW* [2,3] method allows the calculation of an induced magnetic field at the nucleus position $B_{ind}\left(r\right)$ to the applied external magnetic field $B_{0}\left(r\right)$, according to:

(6) $$B_{ind}\left(r\right)=-\sigma \left(r\right)\cdot B_{0}\left(r\right)$$

where σ is the magnetic shielding tensor that proceeds through the calculation of the first-order induced current density $j^{\left(1\right)}\left(r\right)$, which reads:

(7) $$\begin{array}{cc} j^{\left(1\right)}\left(r\right) & =-\sum_{i}^{occ}\left(\phi_{i}^{0}\left(r\right)\nabla \phi_{i}^{1}\left(r\right)+\phi_{i}^{1}\left(r\right)\nabla \phi_{i}^{0}\left(r\right)\right)-\frac{1}{c}n_{0}A\left(r\right)\\ \; & =j_{p}^{\left(1\right)}\left(r\right)+j_{d}^{\left(1\right)}\left(r\right) \end{array}$$

The summation runs over the occupied states and $n_{0}\left(r\right)$ is the unperturbed charge density. In addition, $\phi^{0}$ are the unperturbed Kohn–Sham orbitals and their first-order $\phi^{1}$ counterpart, perturbed due to the external magnetic field. In Equation (7) is given the decomposition of the induced current into the so-called paramagnetic term $j_{p}^{\left(1\right)}\left(r\right)$, which involves the first-order perturbed orbitals, and the diamagnetic term $j_{d}^{\left(1\right)}\left(r\right)$, which depends on the unperturbed charge density. A(r) is the vector potential connected to $B_{0}$ through

(8) $$A\left(r\right)=\frac{1}{2}B_{0}\left(r\right)\times \left(r-r_{G}\right)$$

where $r_{G}$ is the so-called gauge origin. $B_{ind}\left(r\right)$ is finally obtained from the Biot–Savart law:

(9) $$\begin{array}{c} B_{ind}\left(r\right)=\frac{1}{c}\int dr_{G}j^{\left(1\right)}\left(r_{G}\right)\times \frac{r-r_{G}}{|r-r_{G}{|}^{3}} \end{array}$$

Actually, there are connections between our *GIPAW* [2,3] approach and the GIAO [1] method widely used in the quantum-chemical community for the all-electron calculation NMR of molecules. However, it should be recognized that, in *GIPAW* [2,3], the phase required to maintain the translational invariance is carried by the operators, whereas in the GIAO [1] approach, the field-dependent phase is attached to the basis functions and to the occupied electronic orbitals, respectively.

## 3. Method and Computational Details

*GIPAW* pseudopotentials were developed for 21 *d*, elements excluding La which is considered as a part of Lanthanides, using Quantum Espresso version 6.6 [20,21] and they are written in the universal pseudopotential format (UPF) version 2 (pseudopotentials will be available to everybody as UPF2 format, see available dataset). *GIPAW* were developed solving the scalar relativistic wave equation (Koelling–Harmon-like equation) [22] with Rappe–Rabe–Efthimios–Kaxiras–Joannopoulos [23] (RRKJ) form of pseudo-wavefunctions modeled by double projectors and semi-core states. A non-linear core correction (NLCC) [24] was employed for all developed *GIPAW*s. The NLCC allows to avoid the necessity to separate spin-up and spin-down ionic pesudopotentials, treating explicitly the nonlinear exchange and correlation interaction between the core and the valence charge densities. In particular, the spin-polarized configurations are well-described with a single potential. The analysis of logarithmic derivatives, i.e., derivatives of an *l*-state $\frac{d(log\left(\Psi_{l}\left(E\right)\right))}{dE}$ computed for the exact atomic problem and with the *GIPAW* dataset, was computed to verify the presence of highly-localized negative energy ghosts that could affect the quality of pseudopotential. No element presented highly localized negative-energy ghosts and only highly localized positive-energy ghosts are seen in the *d* orbital of Nb Os and Ta, but they are located too high in energy (i.e., 1 Ry for Nb and Os, 4 Ry for Ta) to affect the quality of pseudopotential also, cases with the promotion of one electron in the *d* orbital will be described. All the logarithmic derivatives and NLCC for each pseudopotential are shown in the available data. Considered oxides and nitrides of *d* elements of first-, second- and third-period, which are semiconductors, were fully optimized in Quantum Espresso version 6.6 [20,21] with previously developed *GIPAW* [2,3] of *d* elements for PBE exchange-correlation density functional [25]. The crystal structures were taken from Material Project [26] and are here identified by their mp-code: AgN${}_{3}$, mp-571297; Au${}_{2}$O${}_{3}$, mp-27253; CdO${}_{2}$, mp-2310; Cr${}_{2}$O${}_{3}$, mp-19399; HgO, mp-1224; IrN${}_{2}$, mp-415; Lu${}_{2}$O${}_{3}$, mp-1427; MoO${}_{3}$, mp-18856; Nb${}_{2}$O${}_{5}$, mp-581967; OsO${}_{4}$, mp-540783; PdN${}_{2}$, mp-1103427; PtO${}_{2}$, mp-7868; Re${}_{2}$O${}_{7}$, mp-1016092; Rh${}_{2}$O${}_{3}$, mp-542734; RuO${}_{4}$, mp-554791; Sc${}_{2}$O${}_{3}$, mp-216; Ta${}_{2}$O${}_{5}$, mp-10390; Tc${}_{2}$O${}_{7}$, mp-27485; V${}_{2}$O${}_{5}$, mp-25279; WO${}_{3}$, mp-18773; Y${}_{2}$O${}_{3}$, mp-2652. All above-mentioned structures are fully optimized with PBE exchange-correlation density functional [25]. The geometry optimization relies on Broyden–Fletcher–Goldfarb–Shanno (BFGS) algorithm [27,28,29,30], with force tolerance for the maximum net force on atoms fixed at 10${}^{-6}$ Ry/Angstrom and the total energy at 10${}^{-8}$ Ry. The kinetic energy plane-wave energy cutoff of 100 Ry, and the Gaussian smearing [31] equal to 0.005 eV were chosen for all structures. The Γ-centered k-point meshes are: 3×3×3 for AgN${}_{3}$, and CdO${}_{2}$; 4×2×2 for Au${}_{2}$O${}_{3}$, Cr${}_{2}$O${}_{3}$, and Re${}_{2}$O${}_{7}$; 3×6×6 for HgO; 6×6×6 for IrN${}_{2}$; 4×4×4 for Lu${}_{2}$O${}_{3}$, MoO${}_{3}$, PdN${}_{2}$, RuO${}_{4}$, and WO${}_{3}$; 2×4×4 for Nb${}_{2}$O${}_{5}$, OsO${}_{4}$, and Ta${}_{2}$O${}_{5}$; 6×6×3 for PtO${}_{2}$; 4×4×2 for Rh${}_{2}$O${}_{3}$, and V${}_{2}$O${}_{5}$; 2×2×2 for Sc${}_{2}$O${}_{3}$, and Y${}_{2}$O${}_{3}$; and 6×4×2 for Tc${}_{2}$O${}_{7}$. The computation of NMR shielding tensors was performed with the *GIPAW* module for Quantum Espresso version 6.6 [20,21].

## 4. Results and Discussion

**Figure 1:**
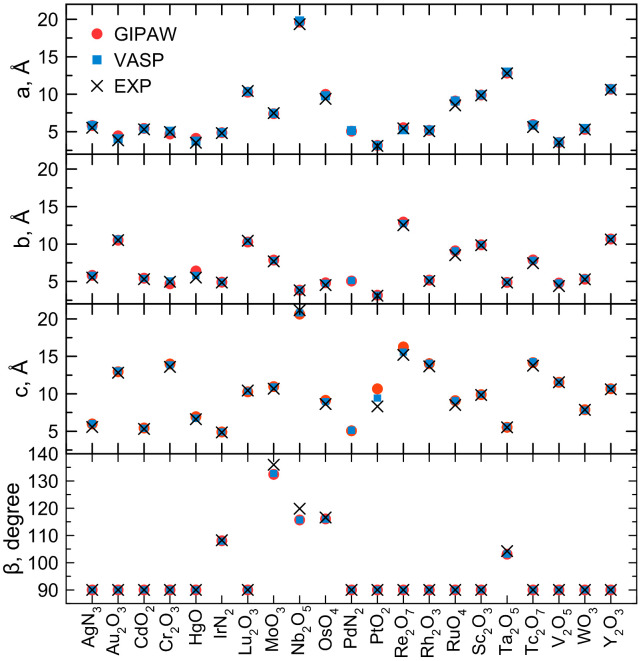
Lattice parameters of 21 fully relaxed unit cells with developed *GIPAW* pseudopotentials (red circles) compared with those optimized with PAW pseudopotentials in VASP code (cyan squares) and experimental ones (black crosses).

In metal transitions, the so-called semi-core states overlap with the *d*-valence states. Thus, we introduced the semi-core states in valence states with a small core region radius, improving the accuracy. For the 3*d* transition metals, the semi-states 3*s* 3*p* 3*d* were chosen as part of the valence partition. The *GIPAW*s for the 4*d* transition metals all contain the 4*s* 4*p* 4*d* semi-states in the valence. For the 5*d* transition metals, the 5*s* 5*p* 5*d* semi-states are contained in the valence of *GIPAW*s. In 5*d* transition metals, 4*f* states were frozen due to the complexity, to be rightly described lying at the same energy range as the 5*s* and 5*p* states. It is noteworthy that, for such elements, the ground-state properties can be described well enough with 4*f* frozen. Meanwhile, for optical properties and *GW* approximation [32,33], this may not be the case. This is the case even for elements such as Au, where the 4*f* electrons lie about 3 Ha below the Fermi level. Instead, for *GIPAW* for 3*d* and 4*d* elements, *GW* approximation [32,33] can also be performed with such pseudopotentials. The ground-state properties of 5*d* elements are not affected by 4*f* orbitals because they are contracted close to the core due to relativistic effects and consequent *jj*-spin orbit coupling. This means that, firstly, for each orbital its orbital angular momentum is coupled with the magnetic angular momentum of the electron located in such an orbital, obtaining an angular momentum *j* and, subsequently, all these *j* are coupled to make the total angular momentum of a heavy atom. We fully relaxed the crystal structures of the oxides and nitrides of the 21 *d* elements of which we developed *GIPAW* pseudopotentials using the PBE DFT functional. The PBE DFT functional is known in the literature to overestimate the unit volume by close to 4–4.5%, but the agreement between the calculated and experimental NMR parameters is generally found to be significantly improved after the DFT optimization of the structure geometry [34]. This motivated our choice to optimize the structure before calculating the NMR parameters. We are aware that all oxides and nitrides of *d* elements are considered strong correlated systems. This means that heuristic DFT alone seems to not be enough to describe correctly the electronic structure spreading the *d* electrons within the unit cell. Usually, DFT+U [35] typically works well for strongly correlated systems localizing the *d* electrons on metals, but previous works showed that the *GIPAW* formalism feasibly describes the transition metals with an heuristic DFT [36,37,38,39]. Thus, we have used a heuristic DFT to fully relax the chosen systems. The fully optimized lattice parameters of such systems agree with those that come from experiments and with the computed structures showed in Material Project [26], see Figure 1 and Tables S1 and S2 in Supporting Information. Such computed structures, reported in Material Project [26], were actually optimized using projector augmented-wave (PAW) pseudopotentials [19] in VASP code [40,41,42,43]. Confident of the quality of the obtained lattice parameters of the oxides and nitrides of the *d* elements, we were able to use the *GIPAW* approach to calculate the total NMR chemical shift (σ) by adopting the Simpson [44] convention for anisotropy and asymmetry and the average value of the *d* elements within the oxide or nitrides are reported in Table 1. We have reported, in Table 1, the average values because, in some structures, the atoms that are in non-equivalent for symmetry positions have different values and in Supporting Information from Tables S3–S23 we reported all atomic positions with corresponding (σ). It is noteworthy that we have obtained negative (σ) in some cases and this is due to the fact that some *d* elements are more shielded with respect to the chosen reference (i.e., TMS) compound. This make us to consider if the evolution of NMR should be focused to find an inorganic compound to set σ. Indeed, here we considered nitrides or oxides that are semiconductors. Thus, our results are not affected by Knight shift, as in metals, due to the high population of *d* electrons at the Fermi level. We also calculated $C_{Q}$ and its average values for the *d* elements within oxides or nitrides, see Table 1, while the value for each atom is reported in Supporting Information. Of note is the impossibility of using predicted NMR spectra from first-principles calculations to solve or refine structures due to the time and cost of the calculation, which poses challenges to a real-time automated solution. To address this problem, machine-learning approaches were introduced to calculate chemical shifts in molecular solids, which reduces computational cost by orders of magnitude while maintaining the accuracy of DFT [45]. All these machine-learning models are trained on first-principles calculations, making the latter of fundamental importance to improve the accuracy of machine-learning models.

## 5. Conclusions

In this work, we developed *GIPAW* pseudopotentials for 21 *d* elements and tested them on their oxides or nitrides. The developed pseudopotentials present semi-core states to increase their flexibility, to be employed in several compounds with feasible approximation, and are free of ghosts states, making them conceptually right. The obtained σ and $C_{Q}$ for the *d* elements seem to be coherent with expected values for such elements, respectively, in their oxides or nitrides. The *GIPAW* pseudopotentials increase the number of compounds for which it will be possible to calculate the NMR parameters with first-principle calculations and subsequently use them to develop machine learning models for real-time refining structure from NMR spectra.

## Figures and Tables

**Table 1:** Average of total chemical shift $\bar{\sigma }$ in ppm and the average of quadrupolar coupling constant ${\bar{C}}_{Q}$ in MHz for each *d* element within their oxides or nitrides.

System	$\bar{\sigma }$	${\bar{C}}_{Q}$	System	$\bar{\sigma }$	${\bar{C}}_{Q}$
AgN${}_{3}$	2542.54	0.04	PtO${}_{2}$	−7557.22	1.20
Au${}_{2}$O${}_{3}$	1115.14	2.45	Re${}_{2}$O${}_{7}$	−1064.07	3.44
CdO${}_{2}$	3322.38	0.09	Rh${}_{2}$O${}_{3}$	−10,829.68	0.05
Cr${}_{2}$O${}_{3}$	−6933.31	−0.07	RuO${}_{4}$	−2968.18	0.02
HgO	6700.46	−15.05	Sc${}_{2}$O${}_{3}$	677.59	−0.25
IrN${}_{2}$	−4132.37	−4.95	Ta${}_{2}$O${}_{5}$	2529.32	2.76
Lu${}_{2}$O${}_{3}$	5317.24	−1.03	Tc${}_{2}$O${}_{7}$	−2249.87	0.43
MoO${}_{3}$	−1196.63	−2.30	V${}_{2}$O${}_{5}$	−1447.62	−0.35
Nb${}_{2}$O${}_{5}$	102.85	−1.06	WO${}_{3}$	752.99	5.18
OsO${}_{4}$	−2337.18	−0.06	Y${}_{2}$O${}_{3}$	1928.05	−0.41
PdN${}_{2}$	−3407.89	−0.37			

## Data Availability

Dataset folder with all generated UPF2 format pseudopotentials will be accessible to the link https://sites.google.com/site/dceresoli/pseudopotentials (accessed on 7 February 2022).

## Supplementary Materials

The following supporting information can be downloaded at: https://www.mdpi.com/article/10.3390/ma15093347/s1, Table S1: Table of lattice parameters of 10 fully-relaxed unit cells with developed *GIPAW* pseudopotentials and those optimized with PAW pseudopotentials in VASP code and the experimental ones; Table S2: Table of lattice parameters of 11 fully-relaxed unit cells with developed *GIPAW* pseudopotentials and those optimized with PAW pseudopotentials in VASP code and the experimental ones; Table S3: Atomic crystallographic positions with vectors lattice in Angstrom of AgN${}_{3}$ with total chemical shift σ in ppm and quadrupolar coupling constant $C_{Q}$ in MHz for each element; Table S4: Atomic crystallographic positions with vectors lattice in Angstrom of Au${}_{2}$O${}_{3}$ with total chemical shift σ in ppm and quadrupolar coupling constant $C_{Q}$ in MHz for each element; Table S5: Atomic crystallographic positions with vectors lattice in Angstrom of CdO${}_{2}$ with total chemical shift σ in ppm and quadrupolar coupling constant $C_{Q}$ in MHz for each element; Table S6: Atomic crystallographic positions with vectors lattice in Angstrom of Cr${}_{2}$O${}_{3}$ with total chemical shift σ in ppm and quadrupolar coupling constant $C_{Q}$ in MHz for each element; Table S7: Atomic crystallographic positions with vectors lattice in Angstrom of HgO with total chemical shift σ in ppm and quadrupolar coupling constant $C_{Q}$ in MHz for each element; Table S8: Atomic crystallographic positions with vectors lattice in Angstrom of IrN${}_{2}$ with total chemical shift σ in ppm and quadrupolar coupling constant $C_{Q}$ in MHz for each element; Table S9: Atomic crystallographic positions with vectors lattice in Angstrom of Lu${}_{2}$O${}_{3}$ with total chemical shift σ in ppm and quadrupolar coupling constant $C_{Q}$ in MHz for each element; Table S10: Atomic crystallographic positions with vectors lattice in Angstrom of MoO${}_{3}$ with total chemical shift σ in ppm and quadrupolar coupling constant $C_{Q}$ in MHz for each element; Table S11: Atomic crystallographic positions with vectors lattice in Angstrom of Nb${}_{2}$O${}_{5}$ with total chemical shift σ in ppm and quadrupolar coupling constant $C_{Q}$ in MHz for each element; Table S12: Atomic crystallographic positions with vectors lattice in Angstrom of OsO${}_{4}$ with total chemical shift σ in ppm and quadrupolar coupling constant $C_{Q}$ in MHz for each element; Table S13: Atomic crystallographic positions with vectors lattice in Angstrom of PdN${}_{2}$ with total chemical shift σ in ppm and quadrupolar coupling constant $C_{Q}$ in MHz for each element; Table S14: Atomic crystallographic positions with vectors lattice in Angstrom of PtO${}_{2}$ with total chemical shift σ in ppm and quadrupolar coupling constant $C_{Q}$ in MHz for each element; Table S15: Atomic crystallographic positions with vectors lattice in Angstrom of Re${}_{2}$O${}_{7}$ with total chemical shift σ in ppm and quadrupolar coupling constant $C_{Q}$ in MHz for each element; Table S16: Atomic crystallographic positions with vectors lattice in Angstrom of Rh${}_{2}$O${}_{3}$ with total chemical shift σ in ppm and quadrupolar coupling constant $C_{Q}$ in MHz for each element; Table S17: Atomic crystallographic positions with vectors lattice in Angstrom of RuO${}_{4}$ with total chemical shift σ in ppm and quadrupolar coupling constant $C_{Q}$ in MHz for each element; Table S18: Atomic crystallographic positions with vectors lattice in Angstrom of Sc${}_{2}$O${}_{3}$ with total chemical shift σ in ppm and quadrupolar coupling constant $C_{Q}$ in MHz for each element; Table S19: Atomic crystallographic positions with vectors lattice in Angstrom of Ta${}_{2}$O${}_{5}$ with total chemical shift σ in ppm and quadrupolar coupling constant $C_{Q}$ in MHz for each element; Table S20: Atomic crystallographic positions with vectors lattice in Angstrom of Tc${}_{2}$O${}_{7}$ with total chemical shift σ in ppm and quadrupolar coupling constant $C_{Q}$ in MHz for each element; Table S21: Atomic crystallographic positions with vectors lattice in Angstrom of V${}_{2}$O${}_{5}$ with total chemical shift σ in ppm and quadrupolar coupling constant $C_{Q}$ in MHz for each element; Table S22: Atomic crystallographic positions with vectors lattice in Angstrom of WO${}_{3}$ with total chemical shift σ in ppm and quadrupolar coupling constant $C_{Q}$ in MHz for each element; Table S23: Atomic crystallographic positions with vectors lattice in Angstrom of Y${}_{2}$O${}_{3}$ with total chemical shift σ in ppm and quadrupolar coupling constant $C_{Q}$ in MHz for each element.
