# Supplementary material for: GIPAW Pseudopotentials of d Elements for Solid-State NMR
Source: Materials (Basel). 2022 May 6;15(9):3347. doi: 10.3390/ma15093347 (PMC9101793; doi:10.3390/ma15093347)
Supplement: Supplementary file 1 [file materials-15-03347-s001.zip › materials-1610478-SI.pdf]

materials

MDPI

Article

Supplementary Information *GIPAW* pseudopotentials of *d*-elements for solid-state NMR

Christian Tantardini [1,2,*], Alexander G. Kvashnin [3] and Davide Ceresoli [4,*]

[1] Department of Chemistry, UiT The Arctic University of Norway, P.O. Box 6050 Langnes, N-9037 Tromsø, Norway
[2] Institute of Solid State Chemistry and Mechanochemistry SB RAS, 630128 Novosibirsk, Russia
[3] Skolkovo Institute of Science and Technology, Bolshoy Boulevard 30, bld. 1, 121205 Moscow, Russian; A.Kvashnin@skoltech.ru
[4] CNR-SCITEC, c/o Dipartimento di Chimica, Università degli studi di Milano, via Golgi 19, 20133 Milano, Italy
* Correspondence: christiantantardini@ymail.com (C.T.); davide.ceresoli@cnr.it (D.C.)

check for updates

Citation: Tantardini, C.; Kvashnin, A. G.; Ceresoli, D. Supplementary Information *GIPAW* pseudopotentials of *d*-elements for solid-state NMR. *Materials* **2022**, *15*, 3347. https://doi.org/10.3390/ma15093347

Academic Editor: Alina Pruna

Received: 8 February 2022
Accepted: 27 April 2022
Published: 6 May 2022

Publisher's Note: MDPI stays neutral with regard to jurisdictional claims in published maps and institutional affiliations.

Copyright: © 2022 by the authors. Licensee MDPI, Basel, Switzerland. This article is an open access article distributed under the terms and conditions of the Creative Commons Attribution (CC BY) license (https://creativecommons.org/licenses/by/4.0/).

Table S1. Table of lattice parameters of 10 fully-relaxed unit cells with developed *GIPAW* pseudopotentials and those optimized with PAW pseudopotentials in VASP code and the experimental ones.

	GIPAW	VASP	EXP	GIPAW	VASP	EXP
	AgN_3	AgN_3	AgN_3 [1]	Au_2O_3	Au_2O_3	Au_2O_3 [2]
a,Å	5.82	5.83	5.52	4.44	4.06	3.84
b, Å	5.82	5.83	5.52	10.51	10.66	10.52
c, Å	5.98	6.03	5.57	12.95	13.05	12.82
alpha	90.00	90.00	90.00	90.00	90.00	90.00
beta	90.00	90.00	90.00	90.00	90.00	90.00
gamma	90.00	90.00	90.00	90.00	90.00	90.00
	CdO_2	CdO_2	CdO_2 [3]	Cr_2O_3	Cr_2O_3	Cr_2O_3 [4]
a,Å	5.44	5.40	5.31	4.69	5.07	4.96
b, Å	5.44	5.40	5.31	4.69	5.07	4.96
c, Å	5.44	5.40	5.31	13.98	13.87	13.59
alpha	90.00	90.00	90.00	90.00	90.00	90.00
beta	90.00	90.00	90.00	90.00	90.00	90.00
gamma	90.00	90.00	90.00	120.00	120.00	120.00
	HgO	HgO	HgO [5]	IrN_2	IrN_2	IrN_2 [6]
a,Å	4.11	3.74	3.52	4.87	4.88	4.81
b, Å	6.40	5.80	5.52	4.91	4.93	4.86
c, Å	6.94	6.74	6.61	4.90	4.92	4.85
alpha	90.00	90.00	90.00	90.00	90.00	90.00
beta	90.00	90.00	90.00	108.05	107.94	108.25
gamma	90.00	90.00	90.00	90.00	90.00	90.00
	Lu_2O_3	Lu_2O_3	Lu_2O_3 [7]	MoO_3	MoO_3	MoO_3 [8]
a,Å	10.27	10.36	10.44	7.40	7.44	7.48
b, Å	10.27	10.36	10.44	7.85	7.80	7.69
c, Å	10.27	10.36	10.44	10.97	10.91	10.68
alpha	90.00	90.00	90.00	90.00	90.00	90.00
beta	90.00	90.00	90.00	132.43	132.75	135.96
gamma	90.00	90.00	90.00	90.00	90.00	90.00
	Nb_2O_5	Nb_2O_5	Nb_2O_5 [9]	OsO_4	OsO_4	OsO_4 [10]
a,Å	19.62	19.79	19.35	9.98	9.79	9.38
b, Å	3.84	3.87	3.82	4.82	4.75	4.52
c, Å	20.64	20.81	21.16	9.13	8.96	8.63
alpha	90.00	90.00	90.00	90.00	90.00	90.00
beta	115.70	115.71	119.83	116.07	115.97	116.60
gamma	90.00	90.00	90.00	90.00	90.00	90.00

Table S2. Table of lattice parameters of 11 fully-relaxed unit cells with developed *GIPAW* pseudopotentials and those optimized with PAW pseudopotentials in VASP code and the experimental ones.

	GIPAW	VASP	EXP	GIPAW	VASP	EXP
	PdN_2	PdN_2	PdN_2	PtO_2	PtO_2	PtO_2 [11]
a,Å	5.06	5.14	-	3.16	3.16	3.10
b, Å	5.06	5.14	-	3.16	3.16	3.10
c, Å	5.06	5.14	-	10.67	9.44	8.32
alpha	90.00	90.00	-	90.00	90.00	90.00
beta	90.00	90.00	-	90.00	90.00	90.00
gamma	90.00	90.00	-	120.00	120.00	120.00
	Re_2O_7	Re_2O_7	Re_2O_7 [12]	Rh_2O_3	Rh_2O_3	Rh_2O_3 [13]
a,Å	5.53	5.56	5.44	5.18	5.21	5.06
b, Å	12.93	12.74	12.51	5.18	5.21	5.06
c, Å	16.26	15.57	15.20	14.03	14.09	13.66
alpha	90.00	90.00	90.00	90.00	90.00	90.00
beta	90.00	90.00	90.00	90.00	90.00	90.00
gamma	90.00	90.00	90.00	120.00	120.00	120.00
	RuO_4	RuO_4	RuO_4 [14]	Sc_2O_3	Sc_2O_3	Sc_2O_3 [15]
a,Å	9.09	9.09	8.51	9.89	9.92	9.84
b, Å	9.09	9.09	8.51	9.89	9.92	9.84
c, Å	9.09	9.09	8.51	9.89	9.92	9.84
alpha	90.00	90.00	90.00	90.00	90.00	90.00
beta	90.00	90.00	90.00	90.00	90.00	90.00
gamma	90.00	90.00	90.00	90.00	90.00	90.00
	Ta_2O_5	Ta_2O_5	Ta_2O_5 [16]	Tc_2O_7	Tc_2O_7	Tc_2O_7 [17]
a,Å	12.82	12.95	12.79	5.95	5.89	5.62
b, Å	4.88	4.93	4.85	7.88	7.82	7.44
c, Å	5.53	5.60	5.53	14.17	14.27	13.76
alpha	90.00	90.00	90.00	90.00	90.00	90.00
beta	103.12	103.17	104.26	90.00	90.00	90.00
gamma	90.00	90.00	90.00	90.00	90.00	90.00
	V_2O_5	V_2O_5	V_2O_5 [18]	WO_3	WO_3	WO_3 [19]
a,Å	3.57	3.62	3.57	5.30	5.41	5.29
b, Å	4.79	4.80	4.38	5.30	5.41	5.29
c, Å	11.53	11.55	11.54	7.88	7.82	7.86
alpha	90.00	90.00	90.00	90.00	90.00	90.00
beta	90.00	90.00	90.00	90.00	90.00	90.00
gamma	90.00	90.00	90.00	90.00	90.00	90.00
	Y_2O_3	Y_2O_3	Y_2O_3 [20]			
a,Å	10.67	10.71	10.60			
b, Å	10.67	10.71	10.60			
c, Å	10.67	10.71	10.60			
alpha	90.00	90.00	90.00			
beta	90.00	90.00	90.00			
gamma	90.00	90.00	90.00			

Table S3. Atomic crystallographic positions with vectors lattice in Angstrom of AgN_3 with total chemical shift σ in ppm and quadrupolar coupling constant C_Q in MHz for each element.

Atom	x	y	z	σ	C_Q
Ag	0.0000000000	0.0000000000	0.2488353368	2542.54	0.0395
Ag	0.0000000000	0.0000000000	0.7511646632	2542.54	0.0395
Ag	0.5000000000	0.5000000000	0.7511646632	2542.54	0.0395
Ag	0.5000000000	0.5000000000	0.2488353368	2542.54	0.0395
N	0.1452725771	0.6452725770	0.0000000000	109.36	0.9795
N	0.3547210402	0.8547210403	0.5000000000	108.43	0.9782
N	0.3547274230	0.1452725771	-0.0000000000	109.36	0.9795
N	0.5000000000	0.0000000000	0.0000000000	-56.21	0.2834
N	0.1452789597	0.3547210402	0.5000000000	108.43	0.9782
N	0.0000000000	0.5000000000	0.0000000000	-56.21	0.2834
N	0.6452789598	0.1452789597	0.5000000000	108.43	0.9782
N	0.8547274229	0.3547274230	0.0000000000	109.36	0.9795
N	0.8547210403	0.6452789598	0.5000000000	108.43	0.9782
N	0.0000000000	0.5000000000	0.5000000000	-55.81	0.2815
N	0.6452725770	0.8547274229	-0.0000000000	109.36	0.9795
N	0.5000000000	0.0000000000	0.5000000000	-55.81	0.2815
V_x	5.816435545	0.000000000	0.000000000		
V_y	0.000000000	5.816435545	0.000000000		
V_z	0.000000000	0.000000000	5.979657770		

Table S4. Atomic crystallographic positions with vectors lattice in Angstrom of Au_2O_3 with total chemical shift σ in ppm and quadrupolar coupling constant C_Q in MHz for each element.

Atom	x	y	z	σ	C_Q
Au	0.7506047966	0.3639592033	0.4548401914	1109.38	2.3886
Au	0.7505997118	0.1358594099	0.0452190196	1112.50	2.4023
Au	0.0083330326	0.3856771012	0.2052914724	1118.74	2.5019
Au	0.0084728125	0.1143106004	0.2947498295	1119.94	2.5048
Au	0.2506047974	0.3639592168	0.9548401909	1109.38	2.3886
Au	0.2505997023	0.1358593889	0.5452189953	1112.49	2.4023
Au	0.5083330175	0.3856771043	0.7052914487	1118.74	2.5019
Au	0.5084728323	0.1143106106	0.7947497872	1119.94	2.5048
Au	0.2505997023	0.8641405961	0.4547810047	1112.49	2.4023
Au	0.2506047974	0.6360408132	0.0451598241	1109.38	2.3886
Au	0.5084728323	0.8856894114	0.2052501978	1119.94	2.5048
Au	0.5083330175	0.6143228657	0.2947085513	1118.74	2.5019
Au	0.7505997118	0.8641405751	0.9547809954	1112.50	2.4023
Au	0.7506047966	0.6360408267	0.5451598086	1109.38	2.3886
Au	0.0084728125	0.8856894216	0.7052501705	1119.94	2.5048
Au	0.0083330326	0.6143228688	0.7947085126	1118.74	2.5019
O	0.4706604234	0.0000000000	0.0000000000	-100.02	-4.0990
O	0.7283148985	0.2499869834	0.2500680570	-98.14	-4.0639
O	0.1030601472	0.2658118017	0.0811322463	69.58	-3.0230
O	0.1034581114	0.2341174665	0.4189458373	68.92	-3.0147
O	0.8561661232	0.0160576796	0.1679963254	68.34	-3.0080
O	0.8560366172	0.4838805796	0.3320462074	67.17	-3.0103
O	0.9706603268	-0.0000000000	0.5000000000	-100.02	-4.0990
O	0.2283149049	0.2499869964	0.7500680402	-98.14	-4.0639
O	0.6030601259	0.2658117746	0.5811322344	69.58	-3.0230
O	0.6034581107	0.2341174633	0.9189458222	68.92	-3.0147
O	0.3561661231	0.0160576744	0.6679962806	68.34	-3.0080
O	0.3560366032	0.4838805868	0.8320461786	67.17	-3.0103
O	0.9708473402	0.5000000000	-0.0000000000	-102.17	-4.0982
O	0.2283149049	0.7500130036	0.2499319598	-98.14	-4.0639
O	0.6034581107	0.7658825367	0.0810541698	68.92	-3.0147
O	0.6030601259	0.7341882254	0.4188677656	69.58	-3.0230
O	0.3560366032	0.5161194132	0.1679538364	67.17	-3.0103
O	0.3561661231	0.9839423316	0.3320037194	68.34	-3.0080
O	0.4708473566	0.5000000000	0.5000000000	-102.17	-4.0982
O	0.7283148985	0.7500130166	0.7499319430	-98.14	-4.0639
O	0.1034581114	0.7658825335	0.5810541627	68.92	-3.0147
O	0.1030601472	0.7341881983	0.9188677457	69.58	-3.0230
O	0.8560366172	0.5161194204	0.6679537926	67.17	-3.0103
O	0.8561661232	0.9839423264	0.8320036896	68.34	-3.0080
V_x	4.444555579	0.000000000	0.000000000		
V_y	0.000000000	10.511051219	-0.015553227		
V_z	0.000000000	-0.019107155	12.946575452		

Table S5. Atomic crystallographic positions with vectors lattice in Angstrom of CdO_2 with total chemical shift σ in ppm and quadrupolar coupling constant C_Q in MHz for each element.

Atom	x	y	z	σ	C_Q
Cd	0.5000000000	-0.0000000000	0.0000000000	3322.38	0.0859
Cd	0.0000000000	0.0000000000	0.5000000000	3322.38	0.0859
Cd	-0.0000000000	0.5000000000	0.0000000000	3322.38	0.0859
Cd	0.5000000000	0.5000000000	0.5000000000	3322.38	0.0859
O	0.0812794084	0.4187205996	0.5812794004	-8.02	7.0380
O	0.4187205996	0.5812794004	0.0812794084	-8.02	7.0380
O	0.5812794004	0.0812794084	0.4187205996	-8.02	7.0380
O	0.9187205996	0.9187205996	0.9187205996	-8.02	7.0380
O	0.0812794084	0.0812794084	0.0812794084	-8.02	7.0380
O	0.4187205996	0.9187205996	0.5812794004	-8.02	7.0380
O	0.5812794004	0.4187205996	0.9187205996	-8.02	7.0380
O	0.9187205996	0.5812794004	0.4187205996	-8.02	7.0380
V_x	5.436482245	0.000000000	0.000000000		
V_y	0.000000000	5.436482245	0.000000000		
V_z	0.000000000	0.000000000	5.436482245		

Table S6. Atomic crystallographic positions with vectors lattice in Angstrom of Cr_2O_3 with total chemical shift σ in ppm and quadrupolar coupling constant C_Q in MHz for each element.

Atom	x	y	z	σ	C_Q
Cr	0.3333333430	0.6666666870	0.0070312626	-6931.01	-0.0681
Cr	0.6666666870	0.3333333430	0.1737402415	-6933.62	-0.0682
Cr	-0.0000000000	-0.0000000000	0.1595430229	-6935.10	-0.0669
Cr	0.3333333430	0.6666666870	0.3262597532	-6934.01	-0.0682
Cr	-0.0000000000	-0.0000000000	0.3404569819	-6935.34	-0.0669
Cr	0.3333333430	0.6666666870	0.5070312699	-6930.76	-0.0681
Cr	0.6666666870	0.3333333430	0.4929687301	-6930.76	-0.0681
Cr	0.0000000000	0.0000000000	0.6595430181	-6935.34	-0.0669
Cr	0.6666666870	0.3333333430	0.6737402168	-6934.01	-0.0682
Cr	0.0000000000	0.0000000000	0.8404569621	-6935.10	-0.0669
Cr	0.3333333430	0.6666666870	0.8262597885	-6933.62	-0.0682
Cr	0.6666666870	0.3333333430	0.9929687324	-6931.01	-0.0681
O	0.3297635629	-0.0000000060	0.2500000059	-221.84	-1.0303
O	0.0000000060	0.3297635689	0.2500000059	-221.84	-1.0303
O	0.6702364011	0.6702364071	0.2500000059	-221.84	-1.0303
O	0.3367974660	0.3333495769	0.0833214601	-222.08	-1.0298
O	0.6666504531	0.0034478980	0.0833214601	-222.08	-1.0298
O	0.9965520810	0.6632025340	0.0833214601	-222.08	-1.0298
O	0.9965520999	0.3333495869	0.5833214282	-222.08	-1.0298
O	0.6666504431	0.6632025430	0.5833214282	-222.08	-1.0298
O	0.3367974570	0.0034478791	0.5833214282	-222.08	-1.0298
O	0.0034478791	0.6666504431	0.4166785418	-222.08	-1.0298
O	0.3333495869	0.3367974570	0.4166785418	-222.08	-1.0298
O	0.6632025430	0.9965520999	0.4166785418	-222.08	-1.0298
O	0.6632025340	0.6666504531	0.9166785549	-222.08	-1.0298
O	0.3333495769	0.9965520810	0.9166785549	-222.08	-1.0298
O	0.0034478980	0.3367974660	0.9166785549	-222.08	-1.0298
O	0.6702364071	0.0000000060	0.7499999941	-221.84	-1.0303
O	-0.0000000060	0.6702364011	0.7499999941	-221.84	-1.0303
O	0.3297635689	0.3297635629	0.7499999941	-221.84	-1.0303
V_x	4.690086188	-0.000000000	0.000000000		
V_y	-2.345043094	4.061733785	-0.000000000		
V_z	0.000000000	0.000000000	13.981492506		

Table S7. Atomic crystallographic positions with vectors lattice in Angstrom of HgO with total chemical shift σ in ppm and quadrupolar coupling constant C_Q in MHz for each element.

Atom	x	y	z	σ	C_Q
Hg	0.7415028137	0.7500000000	0.3795168369	6700.46	-15.0543
Hg	0.2415027987	0.2500000000	0.1204831781	6700.46	-15.0543
Hg	0.2584971863	0.2500000000	0.6204831931	6700.46	-15.0543
Hg	0.7584971863	0.7500000000	0.8795168069	6700.46	-15.0543
O	0.0019791187	0.7500000000	0.1324661481	-75.43	-3.2431
O	0.5019791257	0.2500000000	0.3675338669	-75.43	-3.2431
O	0.9980208743	0.2500000000	0.8675338369	-75.43	-3.2431
O	0.4980208743	0.7500000000	0.6324661631	-75.43	-3.2431
V_x	4.111798301	0.000000000	0.000000000		
V_y	0.000000000	6.401409578	0.000000000		
V_z	0.000000000	0.000000000	6.943959451		

Table S8. Atomic crystallographic positions with vectors lattice in Angstrom of IrN_2 with total chemical shift σ in ppm and quadrupolar coupling constant C_Q in MHz for each element.

Atom	x	y	z	σ	C_Q
Ir	0.7657112105	0.4999716617	0.2779448536	-4132.37	-4.9472
Ir	0.2342887605	-0.0000283673	0.2220551464	-4132.37	-4.9472
Ir	0.2342887605	0.5000283683	0.7220551464	-4132.37	-4.9472
Ir	0.7657112105	1.0000283383	0.7779448536	-4132.37	-4.9472
N	0.6737671571	0.0886374877	0.3413797969	-59.32	1.8633
N	0.3262328429	0.5886375027	0.1586202031	-59.32	1.8633
N	0.3262328429	0.9113624973	0.6586202331	-59.32	1.8633
N	0.6737671571	0.4113624973	0.8413797669	-59.32	1.8633
N	0.8159056364	0.5994971946	0.6974220543	-131.16	1.4674
N	0.1840943336	0.0994972176	0.8025779457	-131.16	1.4674
N	0.1840943336	0.4005027754	0.3025779747	-131.16	1.4674
N	0.8159056364	0.9005028054	0.1974220393	-131.16	1.4674
V_x	4.868755833	0.000000000	-0.004531696		
V_y	0.000000000	4.909749507	0.000000000		
V_z	-1.515686762	0.000000000	4.664866477		

Table S9. Atomic crystallographic positions with vectors lattice in Angstrom of Lu_2O_3 with total chemical shift σ in ppm and quadrupolar coupling constant C_Q in MHz for each element.

Atom	x	y	z	σ	C_Q
Lu	0.0000068562	0.2500045430	0.4665328547	5292.67	-3.5672
Lu	0.0334794625	0.0000012289	0.7499904193	5292.72	-3.5659
Lu	0.7499904193	0.0334794625	0.0000012289	5292.72	-3.5659
Lu	0.4665328547	0.0000068562	0.2500045430	5292.67	-3.5672
Lu	0.2500045430	0.4665328547	0.0000068562	5292.67	-3.5672
Lu	0.0000012289	0.7499904193	0.0334794625	5292.72	-3.5659
Lu	0.4999893974	0.2499926171	0.0334763343	5292.74	-3.5608
Lu	0.4665269367	0.5000011432	0.7500048954	5292.92	-3.5711
Lu	0.7500048954	0.4665269367	0.5000011432	5292.92	-3.5711
Lu	0.0334763343	0.4999893974	0.2499926171	5292.74	-3.5608
Lu	0.2499926171	0.0334763343	0.4999893974	5292.74	-3.5608
Lu	0.5000011432	0.7500048954	0.4665269367	5292.92	-3.5711
Lu	0.7500049373	0.2500002751	0.2500047689	5390.71	6.5951
Lu	0.2500002751	0.2500047689	0.7500049373	5390.71	6.5951
Lu	0.2500047689	0.7500049373	0.2500002751	5390.71	6.5951
Lu	0.2499899590	0.2499899590	0.2499899590	5390.48	6.5929
Lu	0.5000106026	0.7500073829	0.9665236767	5292.74	-3.5608
Lu	0.5334730633	0.4999988568	0.2499951046	5292.92	-3.5711
Lu	0.2499951046	0.5334730633	0.4999988568	5292.92	-3.5711
Lu	0.9665236767	0.5000106026	0.7500073829	5292.74	-3.5608
Lu	0.7500073829	0.9665236767	0.5000106026	5292.74	-3.5608
Lu	0.4999988568	0.2499951046	0.5334730633	5292.92	-3.5711
Lu	-0.0000068562	0.7499954570	0.5334671453	5292.67	-3.5672
Lu	0.9665205485	-0.0000012289	0.2500095807	5292.72	-3.5659
Lu	0.2500095807	0.9665205485	-0.0000012289	5292.72	-3.5659
Lu	0.5334671453	-0.0000068562	0.7499954570	5292.67	-3.5672
Lu	0.7499954570	0.5334671453	-0.0000068562	5292.67	-3.5672
Lu	-0.0000012289	0.2500095807	0.9665205485	5292.72	-3.5659
Lu	0.2499950627	0.7499997249	0.7499952311	5390.71	6.5951
Lu	0.7499997249	0.7499952311	0.2499950627	5390.71	6.5951
Lu	0.7499952311	0.2499950627	0.7499997249	5390.71	6.5951
Lu	0.7500100410	0.7500100410	0.7500100410	5390.48	6.5929
O	0.1524164061	0.8801759756	0.6087783966	-95.19	0.3665
O	0.3475717339	0.8802078208	0.3911233667	-95.23	0.3667
O	0.1524499425	0.1198078475	0.8911520256	-95.20	0.3664
O	0.1197970167	0.1088216041	0.3475725878	-95.21	0.3662
O	0.1088216041	0.3475725878	0.1197970167	-95.21	0.3662
O	0.6087783966	0.1524164061	0.8801759756	-95.19	0.3665
O	0.8802078208	0.3911233667	0.3475717339	-95.23	0.3667
O	0.8911520256	0.1524499425	0.1198078475	-95.20	0.3664
O	0.8801759756	0.6087783966	0.1524164061	-95.19	0.3665
O	0.3911233667	0.3475717339	0.8802078208	-95.23	0.3667
O	0.1198078475	0.8911520256	0.1524499425	-95.20	0.3664
O	0.3475725878	0.1197970167	0.1088216041	-95.21	0.3662
O	0.3475792385	0.6198224817	0.8911711367	-95.22	0.3666
O	0.1524570208	0.6198266793	0.1088292169	-95.21	0.3671
O	0.3475737889	0.3801900653	0.6088777457	-95.22	0.3668
O	0.3801866808	0.3911185475	0.1524681436	-95.20	0.3673
O	0.1524681436	0.3801866808	0.3911185475	-95.20	0.3673
O	0.3801900653	0.6088777457	0.3475737889	-95.22	0.3668
O	0.1088292169	0.1524570208	0.6198266793	-95.21	0.3671
O	0.6198224817	0.8911711367	0.3475792385	-95.22	0.3666
O	0.6088777457	0.3475737889	0.3801900653	-95.22	0.3668
O	0.6198266793	0.1088292169	0.1524570208	-95.21	0.3671
O	0.8911711367	0.3475792385	0.6198224817	-95.22	0.3666
O	0.3911185475	0.1524681436	0.3801866808	-95.20	0.3673
O	0.6524207915	0.3801775183	0.1088288783	-95.22	0.3666
O	0.8475429492	0.3801733207	0.8911707981	-95.21	0.3671
O	0.6524262411	0.6198099347	0.3911222243	-95.22	0.3668
O	0.6198133192	0.6088814225	0.8475318264	-95.20	0.3673
O	0.6088814225	0.8475318264	0.6198133192	-95.20	0.3673
O	0.1088288783	0.6524207915	0.3801775183	-95.22	0.3666
O	0.3801733207	0.8911707981	0.8475429492	-95.21	0.3671
O	0.3911222243	0.6524262411	0.6198099347	-95.22	0.3668
O	0.3801775183	0.1088288783	0.6524207915	-95.22	0.3666
O	0.8911707981	0.8475429492	0.3801733207	-95.21	0.3671
O	0.6198099347	0.3911222243	0.6524262411	-95.22	0.3668
O	0.8475318264	0.6198133192	0.6088814225	-95.20	0.3673
O	0.8475835639	0.1198240244	0.3912215734	-95.19	0.3665
O	0.6524282961	0.1197921792	0.6088766033	-95.23	0.3667
O	0.8475500275	0.8801921525	0.1088479894	-95.20	0.3664
O	0.8802029833	0.8911784109	0.6524274422	-95.21	0.3662
O	0.6524274422	0.8802029833	0.8911784109	-95.21	0.3662
O	0.8801921525	0.1088479894	0.8475500275	-95.20	0.3664
O	0.6088766033	0.6524282961	0.1197921792	-95.23	0.3667
O	0.1198240244	0.3912215734	0.8475835639	-95.19	0.3665
O	0.1088479894	0.8475500275	0.8801921525	-95.20	0.3664
O	0.1197921792	0.6088766033	0.6524282961	-95.23	0.3667
O	0.3912215734	0.8475835639	0.1198240244	-95.19	0.3665
O	0.8911784109	0.6524274422	0.8802029833	-95.21	0.3662
V_x	10.268670937	0.000081670	0.000081670		
V_y	0.000081670	10.268670937	0.000081670		
V_z	0.000081670	0.000081670	10.268670937		

Table S10. Atomic crystallographic positions with vectors lattice in Angstrom of MoO_3 with total chemical shift σ in ppm and quadrupolar coupling constant C_Q in MHz for each element.

Atom	x	y	z	σ	C_Q
Mo	0.4631898717	0.5398726930	0.7129519266	-1196.63	-2.3031
Mo	0.5368101283	0.0398727230	0.7870480734	-1196.63	-2.3031
Mo	0.5368101283	0.4601272770	0.2870481024	-1196.63	-2.3031
Mo	0.4631898717	0.9601273070	0.2129519116	-1196.63	-2.3031
Mo	0.9627496005	0.5398687320	0.2129632747	-1196.63	-2.3039
Mo	0.0372504185	0.0398687620	0.2870367253	-1196.63	-2.3039
Mo	0.0372504185	0.4601312380	0.7870367253	-1196.63	-2.3039
Mo	0.9627496005	0.9601312680	0.7129632747	-1196.63	-2.3039
O	0.2559760885	0.0209516778	0.5007572275	-532.69	-0.2700
O	0.7440239115	0.5209516668	0.9992427725	-532.69	-0.2700
O	0.7440239115	0.9790483332	0.4992427725	-532.69	-0.2700
O	0.2559760885	0.4790483332	0.0007572155	-532.69	-0.2700
O	0.2544989071	0.5208453471	0.4992414369	-532.71	-0.2702
O	0.7455010639	0.0208453631	0.0007585491	-532.71	-0.2702
O	0.7455010639	0.4791546229	0.5007585631	-532.71	-0.2702
O	0.2544989071	0.9791546529	0.9992414369	-532.71	-0.2702
O	0.7542296038	0.9949350308	0.7545131765	-335.55	1.4153
O	0.2457703812	0.4949350008	0.7454868235	-335.55	1.4153
O	0.2457703812	0.0050649842	0.2454868525	-335.55	1.4153
O	0.7542296038	0.5050649692	0.2545131475	-335.55	1.4153
O	0.7548068165	0.5051060869	0.7545141586	-335.50	1.4157
O	0.2451932135	0.0051061019	0.7454858414	-335.50	1.4157
O	0.2451932135	0.4948939131	0.2454858414	-335.50	1.4157
O	0.7548068165	0.9948939131	0.2545141586	-335.50	1.4157
O	-0.0003186670	0.7390729810	0.7442028245	-487.35	-0.5429
O	1.0003186600	0.2390729810	0.7557971755	-487.35	-0.5429
O	1.0003186600	0.2609270190	0.2557971755	-487.35	-0.5429
O	-0.0003186670	0.7609270190	0.2442028245	-487.35	-0.5429
O	0.4888034327	0.7390500995	0.2440874799	-487.31	-0.5426
O	0.5111965373	0.2390500705	0.2559125051	-487.31	-0.5426
O	0.5111965373	0.2609499295	0.7559124901	-487.31	-0.5426
O	0.4888034327	0.7609499005	0.7440875099	-487.31	-0.5426
V_x	7.399921595	0.000000000	-0.014594631		
V_y	0.000000000	7.854724055	0.000000000		
V_z	-7.382519462	0.000000000	8.109404975		

Table S11. Atomic crystallographic positions with vectors lattice in Angstrom of Nb_2O_5 with total chemical shift σ in ppm and quadrupolar coupling constant C_Q in MHz for each element.

Atom	x	y	z	σ	C_Q
Nb	0.8403852901	0.9896705061	0.8344341284	55.51	-1.0974
Nb	0.5786625748	0.4986669938	0.7035403037	30.08	-1.1044
Nb	0.9961492286	0.0077837199	0.7631602489	113.17	-1.1599
Nb	0.8445850745	0.9756817224	0.5667180046	212.94	-2.9258
Nb	0.3067989793	0.0029924778	0.6287878940	110.32	-0.9506
Nb	0.0038507944	0.0077837199	0.2368397811	113.17	-1.1599
Nb	0.8482376602	0.9935500229	0.3035479036	106.87	-1.2767
Nb	0.2660219738	0.4946178321	0.8410828457	97.30	-1.4469
Nb	0.6852480663	-0.0020828362	0.6392903432	20.30	1.0843
Nb	0.3147519337	-0.0020828362	0.3607096568	20.30	1.0843
Nb	0.1517623248	0.9935500229	0.6964520664	106.87	-1.2767
Nb	0.4682356352	-0.0012556506	0.5622151869	-3.81	1.5910
Nb	0.8894756303	0.5129696084	0.0929812847	102.55	-2.2404
Nb	0.4191931607	0.5009956199	0.7708462238	105.30	-1.2257
Nb	0.7349057821	0.4959014499	0.8982584815	114.89	-0.5427
Nb	0.5752973301	0.5299274238	0.9645232922	262.04	-2.8895
Nb	0.4213374552	0.4986669938	0.2964596963	30.08	-1.1044
Nb	-0.0000000000	0.0252180109	0.5000000000	329.21	-3.6313
Nb	0.0000000000	0.2367376619	0.0000000000	-104.36	2.2843
Nb	0.1105243697	0.5129696084	0.9070186853	102.55	-2.2404
Nb	0.5317643348	-0.0012556506	0.4377847831	-3.81	1.5910
Nb	0.1554149255	0.9756817224	0.4332820254	212.94	-2.9258
Nb	0.5808068393	0.5009956199	0.2291537612	105.30	-1.2257
Nb	0.7339779962	0.4946178321	0.1589171393	97.30	-1.4469
Nb	0.1596146949	0.9896705061	0.1655658866	55.51	-1.0974
Nb	0.6932009907	0.0029924778	0.3712121060	110.32	-0.9506
Nb	0.4247026699	0.5299274238	0.0354767308	262.04	-2.8895
Nb	0.2650941879	0.4959014499	0.1017415105	114.89	-0.5427
O	0.8179993134	0.5006526474	0.1318724039	-352.40	-0.1563
O	0.6605052954	0.4978967828	0.0665607685	-352.53	-0.2364
O	0.2455289535	0.9974543597	0.8132043638	-214.21	0.8092
O	0.4228918230	-0.0011349731	0.4617609391	-223.89	0.3513
O	0.9133434024	0.9952863413	0.9390846111	-328.81	0.5737
O	0.9862431208	0.5050602260	0.2064276903	-182.89	0.8277
O	0.5948123435	1.0010187935	0.2588867769	-174.46	0.8753
O	0.9207549919	0.9898862953	0.5363300571	-388.25	-0.5820
O	0.5771081770	-0.0011349731	0.5382390609	-223.89	0.3513
O	0.8346716148	0.4960225175	0.5735095494	-373.03	-0.5793
O	0.6589454140	0.5002198051	0.2013904595	-357.69	-0.2044
O	0.4988361973	0.5020295816	0.8618365044	-341.74	-0.3966
O	0.5737352800	-0.0012393233	0.6708129607	-24.61	-0.6620
O	0.1653284152	0.4960225175	0.4264904206	-373.03	-0.5793
O	0.4051876565	1.0010187935	0.7411132521	-174.46	0.8753
O	0.6595627915	0.4986194907	0.7913024237	-336.30	-0.6806
O	0.3353289098	0.5016175716	0.6529911951	-180.67	0.8725
O	0.4960706670	0.4989270566	0.5855948840	-129.62	-1.0328
O	0.5851049912	1.0061572148	0.9629368740	-378.09	-0.3768
O	0.3410546160	0.5002198051	0.7986095545	-357.69	-0.2044
O	0.0819419091	0.0058635288	0.3266533823	-343.90	-0.3499
O	0.7537941703	0.9993245075	0.7373451036	-329.75	-0.4177
O	0.6569512489	0.4985487800	0.6454756875	-165.40	0.8617
O	0.9236472674	0.0015993666	0.2644811202	-357.60	-0.2627
O	0.1795287699	0.4956181685	0.1530342317	-203.93	0.7877
O	0.7617395176	0.0028209231	0.4659665898	-336.51	-0.4602
O	0.5039293330	0.4989270566	0.4144051160	-129.62	-1.0328
O	0.2379520767	0.0006445219	0.6684040561	-366.12	-0.2876
O	0.3928551459	0.9996192150	0.5890617082	-377.07	0.1017
O	0.1738780251	0.4973345305	0.7231075887	-218.88	0.8082
O	0.1820006716	0.5006526474	0.8681276111	-352.40	-0.1563
O	-0.0000000000	0.4992585539	0.5000000000	-354.18	-0.6017
O	0.9736070568	0.5063504223	0.0596079184	-329.14	0.4294
O	0.4262647200	-0.0012393233	0.3291870393	-24.61	-0.6620
O	0.0866565826	0.9952863413	0.0609153779	-328.81	0.5737
O	0.8185113743	0.5037643538	0.9969515820	-325.93	-0.6212
O	0.9180580989	0.0058635288	0.6733466177	-343.90	-0.3499
O	0.5011637727	0.5020295816	0.1381634956	-341.74	-0.3966
O	0.4148949788	1.0061572148	0.0370631110	-378.09	-0.3768
O	0.0813058163	0.9943734053	0.6027063563	-356.72	-0.2501
O	0.3404372085	0.4986194907	0.2086975613	-336.30	-0.6806
O	0.0856914392	0.9964471979	0.1953851800	-352.26	-0.2542
O	0.9143085758	0.9964471979	0.8046148350	-352.26	-0.2542
O	0.4988159509	0.4997126787	0.2719974107	-368.08	0.5260
O	0.7620479083	0.0006445219	0.3315959439	-366.12	-0.2876
O	0.7544710465	0.9974543597	0.1867956212	-214.21	0.8092
O	0.7468901588	-0.0035211251	0.5971879766	-403.76	-0.1210
O	0.0137568712	0.5050602260	0.7935723247	-182.89	0.8277
O	0.8261220049	0.4973345305	0.2768924113	-218.88	0.8082
O	0.8204712001	0.4956181685	0.8469657533	-203.93	0.7877
O	0.9186941837	0.9943734053	0.3972936437	-356.72	-0.2501
O	0.3430487811	0.4985487800	0.3545243125	-165.40	0.8617
O	0.2531098412	-0.0035211251	0.4028120234	-403.76	-0.1210
O	0.0852157471	0.0062415149	0.8821551111	-188.23	0.8791
O	0.6608069808	0.5000400369	0.9268392034	-387.29	0.1132
O	0.6646710602	0.5016175716	0.3470088339	-180.67	0.8725
O	0.1814886257	0.5037643538	0.0030484260	-325.93	-0.6212
O	0.5011840791	0.4997126787	0.7280025593	-368.08	0.5260
O	0.0263929432	0.5063504223	0.9403920666	-329.14	0.4294
O	0.6071448841	0.9996192150	0.4109382628	-377.07	0.1017
O	0.0792449861	0.9898862953	0.4636699429	-388.25	-0.5820
O	0.2382604524	0.0028209231	0.5340334102	-336.51	-0.4602
O	0.5000000000	0.5089230610	0.0000000000	-365.57	-0.4945
O	0.9147842759	0.0062415149	0.1178448589	-188.23	0.8791
O	0.0763527096	0.0015993666	0.7355188798	-357.60	-0.2627
O	0.3394947046	0.4978967828	0.9334392385	-352.53	-0.2364
O	0.7523456305	0.9972562578	0.8852801044	-206.19	0.6238
O	0.3391930192	0.5000400369	0.0731607966	-387.29	0.1132
O	0.2476543695	0.9972562578	0.1147199256	-206.19	0.6238
O	0.2462058297	0.9993245075	0.2626549264	-329.75	-0.4177
V_x	19.621480124	0.000000000	0.001491035		
V_y	0.000000000	3.843701415	0.000000000		
V_z	-8.951671660	0.000000000	18.598303331		

Table S12. Atomic crystallographic positions with vectors lattice in Angstrom of OsO_4 with total chemical shift σ in ppm and quadrupolar coupling constant C_Q in MHz for each element.

Atom	x	y	z	σ	C_Q
Os	0.4999993101	0.7540354176	0.2499989704	-2337.30	-0.0628
Os	0.5000006899	0.2459645974	0.7500010296	-2337.30	-0.0628
Os	0.0000006221	0.2540324733	0.2499994966	-2337.06	-0.0602
Os	-0.0000006221	0.7459675267	0.7500005034	-2337.06	-0.0602
O	0.1113188017	0.0496518339	0.1975590528	-468.88	0.7504
O	0.3886812436	0.5496517558	0.3024407524	-468.87	0.7508
O	0.8886811683	0.9503481921	0.8024409472	-468.88	0.7504
O	0.6113187864	0.4503482142	0.6975592476	-468.87	0.7508
O	0.6077527560	0.9585511976	0.4111108072	-468.17	0.7613
O	0.3922486298	0.9585500863	0.0888904662	-468.16	0.7611
O	0.3922472440	0.0414488244	0.5888891928	-468.17	0.7613
O	0.6077513702	0.0414499357	0.9111095338	-468.16	0.7611
O	0.6113196977	0.5496513204	0.1975595312	-468.87	0.7508
O	0.8886808339	0.0496511862	0.3024402045	-468.89	0.7506
O	0.3886803323	0.4503486496	0.8024404688	-468.87	0.7508
O	0.1113191361	0.9503488398	0.6975597955	-468.89	0.7506
O	0.1077522813	0.4585504524	0.4111100972	-468.20	0.7615
O	0.8922477489	0.4585506764	0.0888891732	-468.19	0.7615
O	0.8922477187	0.5414495176	0.5888899028	-468.20	0.7615
O	0.1077522511	0.5414492936	0.9111108268	-468.19	0.7615
V_x	9.980530711	0.000005367	-0.007467201		
V_y	0.000002556	4.821342315	0.000000698		
V_z	-4.006547169	-0.000000370	8.204425895		

Table S13. Atomic crystallographic positions with vectors lattice in Angstrom of PdN_2 with total chemical shift σ in ppm and quadrupolar coupling constant C_Q in MHz for each element.

Atom	x	y	z	σ	C_Q
Pd	0.5000000000	0.5000000000	-0.0000000000	-3407.89	-0.3730
Pd	0.5000000000	0.0000000000	0.5000000000	-3407.89	-0.3730
Pd	-0.0000000000	0.5000000000	0.5000000000	-3407.89	-0.3730
Pd	0.0000000000	-0.0000000000	-0.0000000000	-3407.89	-0.3730
N	0.0704848304	0.9295151556	0.4295151846	215.76	-0.3262
N	0.9295151556	0.4295151846	0.0704848304	215.76	-0.3262
N	0.4295151846	0.0704848304	0.9295151556	215.76	-0.3262
N	0.5704848444	0.5704848444	0.5704848444	215.76	-0.3262
N	0.9295151556	0.0704848304	0.5704848444	215.76	-0.3262
N	0.0704848304	0.5704848444	0.9295151556	215.76	-0.3262
N	0.5704848444	0.9295151556	0.0704848304	215.76	-0.3262
N	0.4295151846	0.4295151846	0.4295151846	215.76	-0.3262
V_x	5.064616944	0.000000000	0.000000000		
V_y	0.000000000	5.064616944	0.000000000		
V_z	0.000000000	0.000000000	5.064616944		

Table S14. Atomic crystallographic positions with vectors lattice in Angstrom of PtO_2 with total chemical shift σ in ppm and quadrupolar coupling constant C_Q in MHz for each element.

Atom	x	y	z	σ	C_Q
Pt	0.3333333430	0.6666666870	0.0000435581	-7557.22	1.1975
Pt	0.6666666870	0.3333333430	0.5000435381	-7557.22	1.1975
O	-0.0000000000	-0.0000000000	0.5873010608	255.86	-1.7944
O	-0.0000000000	-0.0000000000	0.0873010388	255.86	-1.7944
O	0.6666666870	0.3333333430	0.9126564140	256.02	-1.7843
O	0.3333333430	0.6666666870	0.4126564140	256.02	-1.7843
V_x	3.161782816	-0.000000000	-0.000000000		
V_y	-1.580891408	2.738184240	0.000000000		
V_z	-0.000000000	0.000000000	10.666756076		

Table S15. Atomic crystallographic positions with vectors lattice in Angstrom of Re_2O_7 with total chemical shift σ in ppm and quadrupolar coupling constant C_Q in MHz for each element.

Atom	x	y	z	σ	C_Q
Re	0.0499739141	0.0355920891	0.6437929397	-1236.60	4.0581
Re	0.6775907943	0.0224646167	0.3591243917	-892.00	2.8246
Re	0.8167963524	0.2080685572	0.1405873747	-884.18	2.7990
Re	0.5501984729	0.2352363668	0.6447870899	-1243.51	4.0667
Re	0.0501984579	0.2647636482	0.3552129101	-1243.51	4.0667
Re	0.3167963524	0.2919314578	0.8594126103	-884.18	2.7990
Re	0.1775907793	0.4775353883	0.6408755793	-892.00	2.8246
Re	0.5499739291	0.4644079149	0.3562070603	-1236.60	4.0581
Re	0.9500260709	0.5355920851	0.8562070603	-1236.60	4.0581
Re	0.3224092347	0.5224646117	0.1408756083	-892.00	2.8246
Re	0.1832036476	0.7080685722	0.3594126103	-884.18	2.7990
Re	0.4498015271	0.7352363818	0.8552129101	-1243.51	4.0667
Re	0.9498015271	0.7647636182	0.1447870749	-1243.51	4.0667
Re	0.6832036476	0.7919314278	0.6405873897	-884.18	2.7990
Re	0.8224092057	0.9775353883	0.8591244207	-892.00	2.8246
Re	0.4500260709	0.9644079149	0.1437929397	-1236.60	4.0581
O	0.5059138729	-0.0089600674	0.2705483324	-179.35	1.2505
O	0.9791420195	0.0042218447	0.9459777243	-511.74	-0.4327
O	-0.0045481450	0.0586000238	0.5430603196	-572.51	1.1374
O	0.5686187984	0.0509067635	0.8587511650	-482.88	0.7891
O	0.2480096444	0.0631849593	0.1409721558	-543.54	1.0832
O	0.7278711243	0.0762546265	0.1424184945	-165.65	1.3137
O	0.7387713958	0.1295595220	0.6698824599	-254.63	0.9196
O	0.2391053342	0.1407166503	0.6695885268	-256.69	0.9117
O	0.2521865221	0.1658953584	0.3562544622	-540.16	1.0963
O	0.7707588624	0.1536319191	0.3560989186	-169.18	1.3192
O	0.0610039692	0.2199425867	0.8566756929	-482.72	0.8028
O	0.4981066374	0.2141603352	0.5437616697	-584.14	1.1599
O	0.4679683839	0.2634403711	0.9469770500	-511.88	-0.4383
O	-0.0073863967	0.2393748907	0.2281715618	-179.56	1.2258
O	0.4926135763	0.2606251243	0.7718284232	-179.56	1.2258
O	0.9679683839	0.2365596439	0.0530229350	-511.88	-0.4383
O	-0.0018933786	0.2858396498	0.4562383303	-584.14	1.1599
O	0.5610039622	0.2800573993	0.1433242781	-482.72	0.8028
O	0.2707588624	0.3463680809	0.6439010814	-169.18	1.3192
O	0.7521865511	0.3341046416	0.6437455378	-540.16	1.0963
O	0.7391053042	0.3592833347	0.3304114432	-256.69	0.9117
O	0.2387713958	0.3704404780	0.3301175401	-254.63	0.9196
O	0.2278711543	0.4237453655	0.8575814755	-165.65	1.3137
O	0.7480096294	0.4368150337	0.8590278372	-543.54	1.0832
O	0.0686187914	0.4490932255	0.1412488350	-482.88	0.7891
O	0.4954518250	0.4413999792	0.4569396504	-572.51	1.1374
O	0.4791420495	0.4957781533	0.0540222977	-511.74	-0.4327
O	1.0059138729	0.5089600764	0.7294516386	-179.35	1.2505
O	0.4940861271	0.4910399536	0.2294516536	-179.35	1.2505
O	0.0208579545	0.5042218467	0.5540222757	-511.74	-0.4327
O	1.0045481750	0.5586000498	0.9569396804	-572.51	1.1374
O	0.4313812016	0.5509067445	0.6412488350	-482.88	0.7891
O	0.7519903707	0.5631849663	0.3590278372	-543.54	1.0832
O	0.2721288457	0.5762546045	0.3575815055	-165.65	1.3137
O	0.2612286192	0.6295595220	0.8301175401	-254.63	0.9196
O	0.7608946958	0.6407166353	0.8304114732	-256.69	0.9117
O	0.7478134489	0.6658953874	0.1437455378	-540.16	1.0963
O	0.2292411376	0.6536318891	0.1439010814	-169.18	1.3192
O	0.9389960378	0.7199425717	0.6433243071	-482.72	0.8028
O	0.5018933926	0.7141603202	0.9562383303	-584.14	1.1599
O	0.5320316161	0.7634403561	0.5530229500	-511.88	-0.4383
O	1.0073864237	0.7393748757	0.2718284232	-179.56	1.2258
O	0.5073863937	0.7606251243	0.7281715768	-179.56	1.2258
O	0.0320316041	0.7365596439	0.4469770500	-511.88	-0.4383
O	1.0018933626	0.7858396798	0.0437616637	-584.14	1.1599
O	0.4389960378	0.7800574283	0.3566757219	-482.72	0.8028
O	0.7292411376	0.8463681109	0.8560989186	-169.18	1.3192
O	0.2478134779	0.8341046126	0.8562544622	-540.16	1.0963
O	0.2608946658	0.8592833647	0.1695885418	-256.69	0.9117
O	0.7612286042	0.8704404780	0.1698824449	-254.63	0.9196
O	0.7721288757	0.9237453955	0.6424185245	-165.65	1.3137
O	0.2519903707	0.9368150337	0.6409721628	-543.54	1.0832
O	0.9313812016	0.9490932555	0.3587511650	-482.88	0.7891
O	0.5045481450	0.9413999502	0.0430603496	-572.51	1.1374
O	0.5208579805	0.9957781533	0.4459776953	-511.74	-0.4327
O	-0.0059138789	1.0089600464	0.7705483614	-179.35	1.2505
V_x	5.526923280	0.000000000	0.000000000		
V_y	0.000000000	12.930866795	0.000000000		
V_z	0.000000000	0.000000000	16.263494170		

Table S16. Atomic crystallographic positions with vectors lattice in Angstrom of Rh_2O_3 with total chemical shift σ in ppm and quadrupolar coupling constant C_Q in MHz for each element.

Atom	x	y	z	σ	C_Q
Rh	0.6666666870	0.3333333430	0.1826301413	-10833.77	0.0448
Rh	0.3333333430	0.6666666870	0.3173698608	-10833.59	0.0448
Rh	-0.0000000000	-0.0000000000	0.1507049531	-10825.85	0.0461
Rh	0.3333333430	0.6666666870	0.0159582872	-10829.52	0.0450
Rh	0.3333333430	0.6666666870	0.5159582877	-10829.69	0.0450
Rh	-0.0000000000	-0.0000000000	0.6507049812	-10825.67	0.0461
Rh	0.6666666870	0.3333333430	0.4840417123	-10829.69	0.0450
Rh	-0.0000000000	-0.0000000000	0.3492950488	-10825.67	0.0461
Rh	-0.0000000000	-0.0000000000	0.8492950169	-10825.85	0.0461
Rh	0.6666666870	0.3333333430	0.9840417248	-10829.52	0.0450
Rh	0.3333333430	0.6666666870	0.8173698437	-10833.77	0.0448
Rh	0.6666666870	0.3333333430	0.6826301392	-10833.59	0.0448
O	0.9664330099	0.6330980362	0.0833323521	370.84	-1.0910
O	0.6666650274	0.0335669721	0.0833323521	370.84	-1.0910
O	0.3669019638	0.3333350026	0.0833323521	370.84	-1.0910
O	0.7003153226	0.7003153194	0.2500000076	370.89	-1.0908
O	-0.0000000032	0.2996846474	0.2500000076	370.89	-1.0908
O	0.2996846506	0.0000000032	0.2500000076	370.89	-1.0908
O	0.6330980401	0.9664330038	0.4166676473	370.84	-1.0910
O	0.3333349927	0.3669019599	0.4166676473	370.84	-1.0910
O	0.0335669782	0.6666650373	0.4166676473	370.84	-1.0910
O	0.3669019599	0.0335669782	0.5833323227	370.84	-1.0910
O	0.6666650373	0.6330980401	0.5833323227	370.84	-1.0910
O	0.9664330038	0.3333349927	0.5833323227	370.84	-1.0910
O	0.2996846474	0.2996846506	0.7499999924	370.89	-1.0908
O	0.0000000032	0.7003153226	0.7499999924	370.89	-1.0908
O	0.7003153194	-0.0000000032	0.7499999924	370.89	-1.0908
O	0.0335669721	0.3669019638	0.9166676629	370.84	-1.0910
O	0.3333350026	0.9664330099	0.9166676629	370.84	-1.0910
O	0.6330980362	0.6666650274	0.9166676629	370.84	-1.0910
V_x	5.183533355	0.000000000	0.000000000		
V_y	-2.591766677	4.489071567	0.000000000		
V_z	-0.000000000	-0.000000000	14.029810312		

Table S17. Atomic crystallographic positions with vectors lattice in Angstrom of RuO_4 with total chemical shift σ in ppm and quadrupolar coupling constant C_Q in MHz for each element.

Atom	x	y	z	σ	C_Q
Ru	0.7500000000	0.5000000000	0.0000000000	-2963.34	0.0323
Ru	0.5000000000	0.5000000000	0.5000000000	-2982.70	-0.0000
Ru	0.5000000000	0.0000000000	0.2500000000	-2963.34	0.0323
Ru	0.0000000000	0.2500000000	0.5000000000	-2963.34	0.0323
Ru	0.0000000000	-0.0000000000	0.0000000000	-2982.70	-0.0000
Ru	0.2500000000	0.5000000000	-0.0000000000	-2963.34	0.0323
Ru	0.5000000000	-0.0000000000	0.7500000000	-2963.34	0.0323
Ru	0.0000000000	0.7500000000	0.5000000000	-2963.34	0.0323
O	0.6084008955	0.8917644690	0.6418734121	-774.37	0.9824
O	0.1082355460	0.3581265579	0.6084008955	-774.37	0.9824
O	0.3917644690	0.8915991045	0.8581265879	-774.37	0.9824
O	0.6082653236	0.3917346764	0.6082653236	-776.61	0.9807
O	0.8917346764	0.1082653236	0.1082653236	-776.61	0.9807
O	0.6084008955	0.1082355460	0.3581265579	-774.37	0.9824
O	0.6082355310	0.1084008955	0.8581265879	-774.37	0.9824
O	0.1084008955	0.8581265879	0.6082355310	-774.37	0.9824
O	0.1082653236	0.1082653236	0.8917346764	-776.61	0.9807
O	0.1418734421	0.6082355310	0.8915991045	-774.37	0.9824
O	0.8917644690	0.3581265579	0.3915991045	-774.37	0.9824
O	0.1418734421	0.3917644690	0.1084008955	-774.37	0.9824
O	0.1082653236	0.8917346764	0.1082653236	-776.61	0.9807
O	0.3581265579	0.6084008955	0.1082355460	-774.37	0.9824
O	0.3915991045	0.8917644690	0.3581265579	-774.37	0.9824
O	0.1082355460	0.6418734121	0.3915991045	-774.37	0.9824
O	0.3917346764	0.6082653236	0.6082653236	-776.61	0.9807
O	0.8915991045	0.8581265879	0.3917644690	-774.37	0.9824
O	0.6418734121	0.6084008955	0.8917644690	-774.37	0.9824
O	0.3581265579	0.3915991045	0.8917644690	-774.37	0.9824
O	0.8581265879	0.3917644690	0.8915991045	-774.37	0.9824
O	0.6082355310	0.8915991045	0.1418734421	-774.37	0.9824
O	0.3917346764	0.3917346764	0.3917346764	-776.61	0.9807
O	0.6082653236	0.6082653236	0.3917346764	-776.61	0.9807
O	0.6418734121	0.3915991045	0.1082355460	-774.37	0.9824
O	0.8915991045	0.1418734421	0.6082355310	-774.37	0.9824
O	0.1084008955	0.1418734421	0.3917644690	-774.37	0.9824
O	0.3915991045	0.1082355460	0.6418734121	-774.37	0.9824
O	0.8581265879	0.6082355310	0.1084008955	-774.37	0.9824
O	0.8917644690	0.6418734121	0.6084008955	-774.37	0.9824
O	0.3917644690	0.1084008955	0.1418734421	-774.37	0.9824
O	0.8917346764	0.8917346764	0.8917346764	-776.61	0.9807
V_x	9.089937250	0.000000000	0.000000000		
V_y	0.000000000	9.089937250	0.000000000		
V_z	0.000000000	0.000000000	9.089937250		

Table S18. Atomic crystallographic positions with vectors lattice in Angstrom of Sc_2O_3 with total chemical shift σ in ppm and quadrupolar coupling constant C_Q in MHz for each element.

Atom	x	y	z	σ	C_Q
Sc	0.0000000000	0.5000000000	-0.0000000000	694.11	1.1189
Sc	0.0000000000	0.0000000000	0.5000000000	694.11	1.1189
Sc	0.5000000000	-0.0000000000	0.0000000000	694.11	1.1189
Sc	-0.0000000000	-0.0000000000	0.0000000000	694.05	1.1203
Sc	0.7497326949	0.2857352705	0.5000334421	672.23	-0.6989
Sc	0.2502485437	0.2137145615	0.5000090730	671.85	-0.7034
Sc	0.2857352705	0.5000334421	0.7497326949	672.23	-0.6989
Sc	0.2137145615	0.5000090730	0.2502485437	671.85	-0.7034
Sc	0.5000334421	0.7497326949	0.2857352705	672.23	-0.6989
Sc	0.5000090730	0.2502485437	0.2137145615	671.85	-0.7034
Sc	0.7497093587	0.2135816411	0.0000636551	671.81	-0.7027
Sc	0.2502697807	0.2858053410	0.0000244943	672.30	-0.6998
Sc	0.2135816411	0.0000636551	0.7497093587	671.81	-0.7027
Sc	0.2858053410	0.0000244943	0.2502697807	672.30	-0.6998
Sc	0.0000636551	0.7497093587	0.2135816411	671.81	-0.7027
Sc	0.0000244943	0.2502697807	0.2858053410	672.30	-0.6998
Sc	0.5000000000	-0.0000000000	0.5000000000	694.35	1.1209
Sc	0.5000000000	0.5000000000	-0.0000000000	694.35	1.1209
Sc	-0.0000000000	0.5000000000	0.5000000000	694.35	1.1209
Sc	0.5000000000	0.5000000000	0.5000000000	694.40	1.1203
Sc	0.2502906413	0.7864183589	-0.0000636551	671.81	-0.7027
Sc	0.7497302193	0.7141946590	-0.0000244943	672.30	-0.6998
Sc	0.7864183589	-0.0000636551	0.2502906413	671.81	-0.7027
Sc	0.7141946590	-0.0000244943	0.7497302193	672.30	-0.6998
Sc	-0.0000636551	0.2502906413	0.7864183589	671.81	-0.7027
Sc	-0.0000244943	0.7497302193	0.7141946590	672.30	-0.6998
Sc	0.2502673051	0.7142647295	0.4999665579	672.23	-0.6989
Sc	0.7497514563	0.7862854385	0.4999909270	671.85	-0.7034
Sc	0.7142647295	0.4999665579	0.2502673051	672.23	-0.6989
Sc	0.7862854385	0.4999909270	0.7497514563	671.85	-0.7034
Sc	0.4999665579	0.2502673051	0.7142647295	672.23	-0.6989
Sc	0.4999909270	0.7497514563	0.7862854385	671.85	-0.7034
O	0.0955707735	0.3584799264	0.1311287663	-211.57	0.3364
O	0.4044328007	0.3584699358	0.8688786233	-211.58	0.3297
O	0.5954648710	0.1414530939	0.8688315029	-211.43	0.3247
O	0.9045002104	0.1414242891	0.1312181300	-211.46	0.3314
O	0.3584799264	0.1311287663	0.0955707735	-211.57	0.3364
O	0.3584699358	0.8688786233	0.4044328007	-211.58	0.3297
O	0.1414530939	0.8688315029	0.5954648710	-211.43	0.3247
O	0.1414242891	0.1312181300	0.9045002104	-211.46	0.3314
O	0.1311287663	0.0955707735	0.3584799264	-211.57	0.3364
O	0.8688786233	0.4044328007	0.3584699358	-211.58	0.3297
O	0.8688315029	0.5954648710	0.1414530939	-211.43	0.3247
O	0.1312181300	0.9045002104	0.1414242891	-211.46	0.3314
O	0.4045206840	0.1414841699	0.3688338510	-211.83	0.3253
O	0.0954466086	0.1415482366	0.6311984446	-211.83	0.3308
O	0.9044046002	0.3585424315	0.6311999946	-212.01	0.3365
O	0.5955380069	0.3584076408	0.3689243145	-211.97	0.3293
O	0.1414841699	0.3688338510	0.4045206840	-211.83	0.3253
O	0.1415482366	0.6311984446	0.0954466086	-211.83	0.3308
O	0.3585424315	0.6311999946	0.9044046002	-212.01	0.3365
O	0.3584076408	0.3689243145	0.5955380069	-211.97	0.3293
O	0.3688338510	0.4045206840	0.1414841699	-211.83	0.3253
O	0.6311984446	0.0954466086	0.1415482366	-211.83	0.3308
O	0.6311999946	0.9044046002	0.3585424315	-212.01	0.3365
O	0.3689243145	0.5955380069	0.3584076408	-211.97	0.3293
O	0.5954792860	0.8585158151	0.6311661790	-211.83	0.3253
O	0.9045534214	0.8584517484	0.3688015854	-211.83	0.3308
O	0.0955954298	0.6414575685	0.3688000354	-212.01	0.3365
O	0.4044619631	0.6415923592	0.6310757155	-211.97	0.3293
O	0.8585158151	0.6311661790	0.5954792860	-211.83	0.3253
O	0.8584517484	0.3688015854	0.9045534214	-211.83	0.3308
O	0.6414575685	0.3688000354	0.0955954298	-212.01	0.3365
O	0.6415923592	0.6310757155	0.4044619631	-211.97	0.3293
O	0.6311661790	0.5954792860	0.8585158151	-211.83	0.3253
O	0.3688015854	0.9045534214	0.8584517484	-211.83	0.3308
O	0.3688000354	0.0955954298	0.6414575685	-212.01	0.3365
O	0.6310757155	0.4044619631	0.6415923592	-211.97	0.3293
O	0.9044292565	0.6415200736	0.8688712187	-211.57	0.3364
O	0.5955671693	0.6415300642	0.1311213617	-211.58	0.3297
O	0.4045350990	0.8585468911	0.1311684821	-211.43	0.3247
O	0.0954998196	0.8585756959	0.8687818550	-211.46	0.3314
O	0.6415200736	0.8688712187	0.9044292565	-211.57	0.3364
O	0.6415300642	0.1311213617	0.5955671693	-211.58	0.3297
O	0.8585468911	0.1311684821	0.4045350990	-211.43	0.3247
O	0.8585756959	0.8687818550	0.0954998196	-211.46	0.3314
O	0.8688712187	0.9044292565	0.6415200736	-211.57	0.3364
O	0.1311213617	0.5955671693	0.6415300642	-211.58	0.3297
O	0.1311684821	0.4045350990	0.8585468911	-211.43	0.3247
O	0.8687818550	0.0954998196	0.8585756959	-211.46	0.3314
V_x	9.894558759	-0.000091112	-0.000091112		
V_y	-0.000091112	9.894558759	-0.000091112		
V_z	-0.000091112	-0.000091112	9.894558759		

Table S19. Atomic crystallographic positions with vectors lattice in Angstrom of Ta_2O_5 with total chemical shift σ in ppm and quadrupolar coupling constant C_Q in MHz for each element.

Atom	x	y	z	σ	C_Q
Ta	0.1408293688	0.7526165543	0.7714563574	2529.32	2.7622
Ta	0.3591706318	0.2526165721	0.7285436451	2529.32	2.7622
Ta	0.3591706318	0.7473834455	0.2285436134	2529.32	2.7622
Ta	0.1408293688	0.2473834279	0.2714563857	2529.32	2.7622
Ta	0.6408293682	0.2526165845	0.7714563566	2529.32	2.7622
Ta	0.8591706312	0.7526165421	0.7285436443	2529.32	2.7622
Ta	0.8591706312	0.2473834157	0.2285436126	2529.32	2.7622
Ta	0.6408293682	0.7473834579	0.2714563849	2529.32	2.7622
O	0.2957029237	0.5679271353	0.8722757245	-99.24	-0.7898
O	0.2042970764	0.0679271637	0.6277242769	-99.24	-0.7898
O	0.2042970769	0.9320728612	0.1277242762	-99.24	-0.7898
O	0.2957029242	0.4320728325	0.3722757236	-99.24	-0.7898
O	0.0000000010	0.1098983592	0.2499999985	-224.98	-0.2314
O	-0.0000000010	0.8901016628	0.7500000015	-224.98	-0.2314
O	0.3881151775	0.0509625369	0.0156100572	-220.68	0.2181
O	0.1118848195	0.5509625363	0.4843899434	-220.68	0.2181
O	0.1118848195	0.4490374702	0.9843899415	-220.68	0.2181
O	0.3881151775	0.9490374712	0.5156100573	-220.68	0.2181
O	0.7957029231	0.0679271608	0.8722757238	-99.24	-0.7898
O	0.7042970758	0.5679271385	0.6277242764	-99.24	-0.7898
O	0.7042970763	0.4320728357	0.1277242755	-99.24	-0.7898
O	0.7957029236	0.9320728583	0.3722757231	-99.24	-0.7898
O	0.5000000010	0.6098983302	0.2499999985	-224.98	-0.2314
O	0.4999999990	0.3901016398	0.7500000015	-224.98	-0.2314
O	0.8881151725	0.5509625298	0.0156100565	-220.68	0.2181
O	0.6118848225	0.0509625438	0.4843899427	-220.68	0.2181
O	0.6118848225	0.9490374781	0.9843899408	-220.68	0.2181
O	0.8881151725	0.4490374637	0.5156100566	-220.68	0.2181
V_x	12.820581888	0.000000059	0.008537863		
V_y	0.000000023	4.877946217	-0.000000007		
V_z	-1.258918400	-0.000000014	5.387104323		

Table S20. Atomic crystallographic positions with vectors lattice in Angstrom of Tc_2O_7 with total chemical shift σ in ppm and quadrupolar coupling constant C_Q in MHz for each element.

Atom	x	y	z	σ	C_Q
Tc	0.8066899657	0.4885814018	0.6034021642	-2249.87	0.4271
Tc	0.3066899357	0.5114185982	0.8965978358	-2249.87	0.4271
Tc	0.6933100343	0.9885814018	0.3965978658	-2249.87	0.4271
Tc	0.1933100493	0.0114185852	0.1034021492	-2249.87	0.4271
Tc	0.1933100493	0.5114185982	0.3965978658	-2249.87	0.4271
Tc	0.6933100343	0.4885814018	0.1034021492	-2249.87	0.4271
Tc	0.3066899357	0.0114185852	0.6034021642	-2249.87	0.4271
Tc	0.8066899657	0.9885814018	0.8965978358	-2249.87	0.4271
O	0.5902686407	0.6263259347	0.5857744453	-713.64	-0.9957
O	0.0902686407	0.3736740353	0.9142255547	-713.64	-0.9957
O	0.9097313593	0.1263259567	0.4142255547	-713.64	-0.9957
O	0.4097313593	0.8736740653	0.0857744453	-713.64	-0.9957
O	0.4097313593	0.3736740353	0.4142255547	-713.64	-0.9957
O	0.9097313593	0.6263259347	0.0857744453	-713.64	-0.9957
O	0.0902686407	0.8736740653	0.5857744453	-713.64	-0.9957
O	0.5902686407	0.1263259567	0.9142255547	-713.64	-0.9957
O	0.0000000000	-0.0000000000	-0.0000000000	-177.22	1.7940
O	0.5000000000	-0.0000000000	0.5000000000	-177.22	1.7940
O	0.5000000000	0.5000000000	-0.0000000000	-177.22	1.7940
O	0.0000000000	0.5000000000	0.5000000000	-177.22	1.7940
O	0.7003605625	0.7892833352	0.8861694653	-720.84	-0.9862
O	0.2003605475	0.2107166948	0.6138305347	-720.84	-0.9862
O	0.7996394375	0.2892833052	0.1138305117	-720.84	-0.9862
O	0.2996394675	0.7107166648	0.3861694953	-720.84	-0.9862
O	0.2996394675	0.2107166948	0.1138305117	-720.84	-0.9862
O	0.7996394375	0.7892833352	0.3861694953	-720.84	-0.9862
O	0.2003605475	0.7107166648	0.8861694653	-720.84	-0.9862
O	0.7003605625	0.2892833052	0.6138305347	-720.84	-0.9862
O	0.9418592394	0.0413474684	0.7954651930	-752.41	-0.9409
O	0.4418592394	0.9586525576	0.7045348070	-752.41	-0.9409
O	0.5581407606	0.5413474424	0.2045348360	-752.41	-0.9409
O	0.0581407676	0.4586525286	0.2954651640	-752.41	-0.9409
O	0.0581407676	0.9586525576	0.2045348360	-752.41	-0.9409
O	0.5581407606	0.0413474684	0.2954651640	-752.41	-0.9409
O	0.4418592394	0.4586525286	0.7954651930	-752.41	-0.9409
O	0.9418592394	0.5413474424	0.7045348070	-752.41	-0.9409
V_x	5.949893651	0.000000000	0.000000000		
V_y	0.000000000	7.878431267	0.000000000		
V_z	0.000000000	0.000000000	14.170062543		

Table S21. Atomic crystallographic positions with vectors lattice in Angstrom of V_2O_5 with total chemical shift σ in ppm and quadrupolar coupling constant C_Q in MHz for each element.

Atom	x	y	z	σ	C_Q
V	0.0000000000	0.3899109505	0.6482653330	-1447.62	-0.3493
V	0.5000000000	0.6100890785	0.8517346670	-1447.62	-0.3493
V	0.5000000000	0.6100890785	0.1482653180	-1447.62	-0.3493
V	0.0000000000	0.3899109505	0.3517346970	-1447.62	-0.3493
O	0.5000000000	0.4926626260	0.6814365967	-261.93	-0.9874
O	0.0000000000	0.5073374030	0.8185634033	-261.93	-0.9874
O	0.0000000000	0.5073374030	0.1814365817	-261.93	-0.9874
O	0.5000000000	0.4926626260	0.3185634033	-261.93	-0.9874
O	0.0000000000	0.0587383317	0.3563247267	-863.14	-0.6045
O	0.5000000000	0.9412616723	0.1436752733	-863.14	-0.6045
O	0.5000000000	0.9412616723	0.8563247267	-863.14	-0.6045
O	0.5000000000	0.5002171728	0.0000000000	-567.10	-1.2239
O	0.0000000000	0.4997828272	0.5000000000	-567.10	-1.2239
O	0.0000000000	0.0587383317	0.6436752733	-863.14	-0.6045
V_x	3.566211263	0.000000000	0.000000000		
V_y	0.000000000	4.791472504	0.000000000		
V_z	0.000000000	0.000000000	11.533825939		

Table S22. Atomic crystallographic positions with vectors lattice in Angstrom of WO_3 with total chemical shift σ in ppm and quadrupolar coupling constant C_Q in MHz for each element.

Atom	x	y	z	σ	C_Q
W	0.0000000000	0.5000000000	0.2185355575	752.99	5.1767
W	0.0000000000	0.5000000000	0.7185355575	752.99	5.1767
W	0.5000000000	0.0000000000	0.2814644425	752.99	5.1767
W	0.5000000000	0.0000000000	0.7814644425	752.99	5.1767
O	0.2633721485	0.2633721485	0.7500000000	-230.70	0.6438
O	0.7633721775	0.7633721775	0.2500000000	-230.70	0.6438
O	0.2633721485	0.7366278225	0.2500000000	-230.70	0.6438
O	0.2366278365	0.2366278365	0.2500000000	-230.70	0.6438
O	0.7633721775	0.2366278365	0.7500000000	-230.70	0.6438
O	0.7366278225	0.7366278225	0.7500000000	-230.70	0.6438
O	0.2366278365	0.7633721775	0.7500000000	-230.70	0.6438
O	0.7366278225	0.2633721485	0.2500000000	-230.70	0.6438
O	0.0000000000	0.5000000000	0.9969324199	-339.07	-0.0343
O	0.5000000000	0.0000000000	0.5030675801	-339.07	-0.0343
O	0.0000000000	0.5000000000	0.4969324499	-339.07	-0.0343
O	0.5000000000	0.0000000000	0.0030675531	-339.07	-0.0343
V_x	5.303352239	0.000000000	0.000000000		
V_y	0.000000000	5.303352239	0.000000000		
V_z	0.000000000	0.000000000	7.879632196		

Table S23. Atomic crystallographic positions with vectors lattice in Angstrom of Y_2O_3 with total chemical shift σ in ppm and quadrupolar coupling constant C_Q in MHz for each element.

Atom	x	y	z	σ	C_Q
Y	0.2501036251	0.2170993921	0.4999968066	1916.78	-1.4667
Y	0.2826985160	0.5000174372	0.7498607538	1916.98	-1.4618
Y	0.4999968066	0.2501036251	0.2170993921	1916.78	-1.4667
Y	0.2170993921	0.4999968066	0.2501036251	1916.78	-1.4667
Y	0.5000174372	0.7498607538	0.2826985160	1916.98	-1.4618
Y	0.7498607538	0.2826985160	0.5000174372	1916.98	-1.4618
Y	0.2501373667	0.2827108888	-0.0000011625	1917.12	-1.4619
Y	0.2170434680	0.0000226436	0.7498551142	1916.76	-1.4626
Y	-0.0000011625	0.2501373667	0.2827108888	1917.12	-1.4619
Y	0.2827108888	-0.0000011625	0.2501373667	1917.12	-1.4619
Y	0.0000226436	0.7498551142	0.2170434680	1916.76	-1.4626
Y	0.7498551142	0.2170434680	0.0000226436	1916.76	-1.4626
Y	-0.0000000000	-0.0000000000	0.5000000000	1961.31	2.7647
Y	0.0000000000	0.0000000000	0.0000000000	1961.30	2.7675
Y	0.5000000000	-0.0000000000	-0.0000000000	1961.31	2.7647
Y	-0.0000000000	0.5000000000	-0.0000000000	1961.31	2.7647
Y	0.7498626333	0.7172891112	0.0000011625	1917.12	-1.4619
Y	0.7829565320	-0.0000226436	0.2501448858	1916.76	-1.4626
Y	0.0000011625	0.7498626333	0.7172891112	1917.12	-1.4619
Y	0.7172891112	0.0000011625	0.7498626333	1917.12	-1.4619
Y	-0.0000226436	0.2501448858	0.7829565320	1916.76	-1.4626
Y	0.2501448858	0.7829565320	-0.0000226436	1916.76	-1.4626
Y	0.7498963749	0.7829006079	0.5000031934	1916.78	-1.4667
Y	0.7173014840	0.4999825628	0.2501392462	1916.98	-1.4618
Y	0.5000031934	0.7498963749	0.7829006079	1916.78	-1.4667
Y	0.7829006079	0.5000031934	0.7498963749	1916.78	-1.4667
Y	0.4999825628	0.2501392462	0.7173014840	1916.98	-1.4618
Y	0.2501392462	0.7173014840	0.4999825628	1916.98	-1.4618
Y	0.5000000000	0.5000000000	0.0000000000	1961.68	2.7690
Y	0.5000000000	0.5000000000	0.5000000000	1961.60	2.7633
Y	0.0000000000	0.5000000000	0.5000000000	1961.68	2.7690
Y	0.5000000000	0.0000000000	0.5000000000	1961.68	2.7690
O	0.4017136693	0.3592795451	0.8700777365	-159.87	0.3196
O	0.5982534749	0.1407473419	0.8700480851	-159.83	0.3166
O	0.9016459018	0.1407371287	0.1299571461	-159.83	0.3200
O	0.8700480851	0.5982534749	0.1407473419	-159.83	0.3166
O	0.3592795451	0.8700777365	0.4017136693	-159.87	0.3196
O	0.3592374423	0.1298994264	0.0983965811	-159.85	0.3222
O	0.1298994264	0.0983965811	0.3592374423	-159.85	0.3222
O	0.1407473419	0.8700480851	0.5982534749	-159.83	0.3166
O	0.1299571461	0.9016459018	0.1407371287	-159.83	0.3200
O	0.1407371287	0.1299571461	0.9016459018	-159.83	0.3200
O	0.8700777365	0.4017136693	0.3592795451	-159.87	0.3196
O	0.0983965811	0.3592374423	0.1298994264	-159.85	0.3222
O	0.0982434799	0.1407727324	0.6299450373	-159.91	0.3200
O	0.9015970189	0.3592513028	0.6299536863	-159.97	0.3223
O	0.5982819951	0.3592302364	0.3700825044	-159.92	0.3189
O	0.6299536863	0.9015970189	0.3592513028	-159.97	0.3223
O	0.4016352869	0.1407354877	0.3700571989	-159.93	0.3165
O	0.6299450373	0.0982434799	0.1407727324	-159.91	0.3200
O	0.3592302364	0.3700825044	0.5982819951	-159.92	0.3189
O	0.3700825044	0.5982819951	0.3592302364	-159.92	0.3189
O	0.3592513028	0.6299536863	0.9015970189	-159.97	0.3223
O	0.3700571989	0.4016352869	0.1407354877	-159.93	0.3165
O	0.1407354877	0.3700571989	0.4016352869	-159.93	0.3165
O	0.1407727324	0.6299450373	0.0982434799	-159.91	0.3200
O	0.9017565121	0.8592272826	0.3700549627	-159.91	0.3200
O	0.0984029731	0.6407486972	0.3700463137	-159.97	0.3223
O	0.4017180049	0.6407697636	0.6299174956	-159.92	0.3189
O	0.3700463137	0.0984029731	0.6407486972	-159.97	0.3223
O	0.8592272826	0.3700549627	0.9017565121	-159.91	0.3200
O	0.8592645273	0.6299428011	0.5983647131	-159.93	0.3165
O	0.6299428011	0.5983647131	0.8592645273	-159.93	0.3165
O	0.6407486972	0.3700463137	0.0984029731	-159.97	0.3223
O	0.6299174956	0.4017180049	0.6407697636	-159.92	0.3189
O	0.6407697636	0.6299174956	0.4017180049	-159.92	0.3189
O	0.3700549627	0.9017565121	0.8592272826	-159.91	0.3200
O	0.5983647131	0.8592645273	0.6299428011	-159.93	0.3165
O	0.5982863307	0.6407204549	0.1299222635	-159.87	0.3196
O	0.4017465251	0.8592526731	0.1299519149	-159.83	0.3166
O	0.0983540902	0.8592628863	0.8700428539	-159.83	0.3200
O	0.1299519149	0.4017465251	0.8592526731	-159.83	0.3166
O	0.9016034109	0.6407625577	0.8701005736	-159.85	0.3222
O	0.1299222635	0.5982863307	0.6407204549	-159.87	0.3196
O	0.8592628863	0.8700428539	0.0983540902	-159.83	0.3200
O	0.8700428539	0.0983540902	0.8592628863	-159.83	0.3200
O	0.8592526731	0.1299519149	0.4017465251	-159.83	0.3166
O	0.8701005736	0.9016034109	0.6407625577	-159.85	0.3222
O	0.6407625577	0.8701005736	0.9016034109	-159.85	0.3222
O	0.6407204549	0.1299222635	0.5982863307	-159.87	0.3196
V_x	10.672742819	0.000204190	0.000204190		
V_y	0.000204190	10.672742819	0.000204190		
V_z	0.000204190	0.000204190	10.672742819		

References

1. Hou, D.; Zhang, F.; Ji, C.; Hannon, T.; Zhu, H.; Wu, J.; Levitas, V.I.; Ma, Y. Phase transition and structure of silver azide at high pressure. *Journal of Applied Physics* **2011**, *110*, 023524.
2. Jones, P.G.; Rumpel, H.; Schwarzmann, E.; Sheldrick, G.M.; Paulus, H. Gold(III) oxide. *Acta Cryst B* **1979**, *35*, 1435–1437.
3. Hoffman, C.W.W.; Ropp, R.C.; Mooney, R.W. Preparation, Properties and Structure of Cadmium Peroxide. *J. Am. Chem. Soc.* **1959**, *81*, 3830–3834.
4. Hill, A.H.; Harrison, A.; Dickinson, C.; Zhou, W.; Kockelmann, W. Crystallographic and magnetic studies of mesoporous eskolaite, Cr2O3. *Microporous and Mesoporous Materials* **2010**, *130*, 280–286.
5. Aurivillius, K.; Rossotti, H.; Theorell, H.; Thorell, B. The Crystal Structure of Mercury(II)oxide Studied by X-Ray and Neutron Diffraction Methods. *Acta Chem. Scand.* **1956**, *10*, 852–866.
6. Yu, R.; Zhan, Q.; De Jonghe, L.C. Crystal Structures of and Displacive Transitions in OsN_2, IrN_2, RuN_2, and RhN_2. *Angewandte Chemie International Edition* **2007**, *46*, 1136–1140.
7. Krsmanovic Whiffen, R.M.; A., Z.; Speghini, A.; Brik, M.G.; Bartova, B.; Bettinelli, M.; Dramicanin, M.D. Structural and spectroscopic studies of Eu^{3+} doped Lu_2O_3-Gd_2O_3 solid solutions. *Optical Materials* **2014**, *36*, 1083–1091.
8. A new modification of ReO_3-type MoO_3 and the deuterated intercalation compound from which it is derived: $D_{0.99}M0O_3$.
9. Gatehouse, B.M.; Wadsley, A.D. The crystal structure of the high temperature form of niobium pentoxide. *Acta Cryst* **1964**, *17*, 1545–1554.
10. Ueki, T.; Zalkin, A.; Templeton, D.H. The crystal structure of osmium tetroxide. *Acta Cryst* **1965**, *19*, 157–160.
11. HOEKSTRA, H.R.; SIEGEL, S.; GALLAGHER, F.X. Reaction of Platinum Dioxide with Some Metal Oxides; 1971; Vol. 98, pp. 39–53.
12. Krebs, B.; Mueller, A.; Beyer, H.H. Crystal structure of rhenium(VII) oxide. *Inorg. Chem.* **1969**, *8*, 436–443.
13. Umemoto, K.; Wentzcovitch, R.M. Effect of the d electrons on phase transitions in transition-metal sesquioxides. *Phys Chem Minerals* **2011**, *38*, 387–395.
14. Ly, F.; Sheikin, I.; Grenier, B.; Huxley, A.D. Magnetic Field-Induced Superconductivity in the Ferromagnet URhGe. *Science* **2005**, *309*, 1343–1346.
15. Schleid, T.; Meyer, G. Single crystals of rare earth oxides from reducing halide melts. *Journal of the Less Common Metals* **1989**, *149*, 73–80.
16. Zibrov, I.P.; Filonenko, V.P.; Sundberg, M.; Werner, P.E. Structures and phase transitions of B-Ta_2O_5 and Z-Ta_2O_5: two high-pressure forms of Ta_2O_5. *Acta Cryst B* **2000**, *56*, 659–665.
17. Krebs, B. Technetium(VII)-oxid: Ein Übergangsmetalloxid mit Molekülstruktur im festen Zustand. *Zeitschrift für anorganische und allgemeine Chemie* **1971**, *380*, 146–159.
18. Shklover, V.; Haibach, T.; Ried, F.; Nesper, R.; Novak, P. Crystal Structure of the Product of Mg^{2+} Insertion into V_2O_5 Single Crystals. *Journal of Solid State Chemistry* **1996**, *123*, 317–323.
19. Locherer, K.R.; Swainson, I.P.; Salje, E.K.H. Transition to a new tetragonal phase of WO$\less$sub$\greater$3$\less$/sub$\greater$: crystal structure and distortion parameters. *J. Phys.: Condens. Matter* **1999**, *11*, 4143–4156.
20. Coduri, M.; Scavini, M.; Allieta, M.; Brunelli, M.; Ferrero, C. Defect Structure of Y-Doped Ceria on Different Length Scales. *Chem. Mater.* **2013**, *25*, 4278–4289.
